# E3 Ubiquitin Ligase Ring Finger Protein 2 Alleviates Cerebral Ischemia–Reperfusion Injury by Stabilizing Mesencephalic Astrocyte‐Derived Neurotrophic Factor Through Monoubiquitination

**DOI:** 10.1111/cns.70136

**Published:** 2024-11-30

**Authors:** Yujun Shen, Jinfeng Wang, Junxing Liang, Ying Chen, Xueyan Wu, Zhenhua Ren, Jiangning Zhou, Lijie Feng, Yuxian Shen

**Affiliations:** ^1^ School of Basic Medical Sciences Anhui Medical University Hefei China; ^2^ Biopharmaceutical Research Institute Anhui Medical University Hefei China; ^3^ Anhui Provincial Key Laboratory for Brain Bank Construction and Resource Utilization Anhui Medical University Hefei China

**Keywords:** ischemia stroke, ischemia–reperfusion injury, mesencephalic astrocyte‐derived neurotrophic factor, monoubiquitination, ring finger protein 2

## Abstract

**Aim:**

Cerebral ischemic stroke (IS) is one of the leading causes of morbidity and mortality globally. However, the mechanisms underlying IS injury remain poorly understood. Ring finger protein 2 (RNF2), the member of the polycomb family (PcG), has been implicated in diverse biological and pathological conditions. However, whether RNF2 plays a role in IS progression is not clarified. This study aims to investigate the potential effects of RNF2 on IS.

**Methods:**

The effects of RNF2 were studied in human postmortem IS brains, a rat model of IS, tunicamycin (TM)‐induced mouse neuroblastoma neuro2a (N2a) cells, and oxygen–glucose deprivation/reperfusion (OGD/R)‐induced SH‐SY5Y cells.

**Results:**

Here, we demonstrated that RNF2 was markedly upregulated both in human postmortem IS brains and ischemic rat brains and RNF2 overexpression alleviated brain injury induced by middle cerebral artery occlusion by reducing neuron apoptosis. Mechanistically, we found that RNF2 is an E3 ubiquitin ligase for the mesencephalic astrocyte‐derived neurotrophic factor (MANF), which confers protection against brain ischemia. RNF2 interacted with MANF and promoted the monoubiquitination of MANF, consequently facilitating its stability and nuclear localization.

**Conclusion:**

Collectively, RNF2 is identified as a critical inhibitor of IS injury by stabilizing MANF through monoubiquitination, suggesting that RNF2 is a potential therapeutic target for IS.

## Graphical abstract

RNF2 was upregulated in the ischemia–reperfusion brain, as well as in nerve cells treated with OGD/R or TM. RNF2 inhibited cell apoptosis. RNF2 promoted MANF nuclear transport. RNF2 monoubiquitinated MANF and stabilized MANF protein level. RNF2 inhibits cell apoptosis dependently on MANF, which can be reversed by recombinant human MANF.

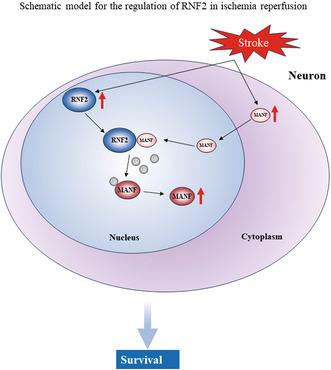

## 1 Introduction

Cerebral stroke is the leading cause of morbidity and mortality worldwide. IS, the most common type of stroke, is an acute cerebrovascular disease characterized by a sudden interruption of blood flow to the brain tissue, subsequent irreversible injury of neurons [1, 2], and loss of neurological function [2]. Reperfusion of the ischemic brain can cause a detrimental secondary brain injury, called cerebral ischemia–reperfusion (I/R) injury, leading to further brain damage and dysfunction [3]. Intravenous thrombolysis with recombinant tissue plasminogen activator or endovascular therapy (mechanical thrombectomy) is the only currently available therapeutic approach in acute IS, but the narrow therapeutic window and side effects limit their clinical application [4]. As such, unraveling the mechanism and identifying the novel factors underlying the pathology of IS is beneficial for the development of efficacious therapies for this life threatening disease.

Cerebral ischemia is a severe form of metabolic stress that interferes with most biochemical and molecular biology pathways [5, 6]. Up to now, it is generally accepted that Endoplasmic reticulum stress occurs during cerebral ischemia [7, 8]. ER stress‐associated proteins, such as GRP78, PERK, and CHOP, in the penumbra zone is related to brain damage because of the disturbance in ER homeostasis [9, 10]. Our preliminary study showed that the newly neurotrophic factor MANF is an ER stress‐sensitive protein and involved in several biological and pathological processes [11, 12]. It was found that MANF was predominantly expressed in neurons and exerted neuroprotection against cerebral I/R injury by inhibiting neuron apoptosis [11, 13], reducing glia activation and inflammation [14, 15] and maintaining ER homeostasis [16, 17]. Thus, regulation of MANF level offers an interesting mechanism to rescue neurons from ischemia injury. Until now, several mechanisms for the regulation of MANF mRNA expression have been established. For example, our previous study uncovers that XBP1s predominantly bound to ERSE I, one of ER stress‐responsive elements (ERSEs) in the promoter of MANF, and promoted MANF transcription [18]. However, few regulatory mechanisms of MANF in the post‐translational modification are clarified till now.

RNF2 (also known as ding, Ring1B, or Ring2) is a member of the polycomb group (PcG) of proteins [19], which form chromatin‐modifying complexes essential for embryonic development and stem cell renewal, which are commonly deregulated in cancer [20, 21, 22, 23]. RNF2 contains a RING finger domain and functions as an E3 ubiquitin ligase for monoubiquitination of histone H2A at lysine 119 [24, 25]. RNF2 has been implicated in a variety of normal physiological processes as well as pathological conditions, such as embryonic development [26, 27], immune surveillance [28], cancer metastasis, and inflammation [19, 29, 30]. However, whether RNF2 plays a role in cerebral IS injury has never been clarified. Previous research has reported that PcG proteins are upregulated in ischemic‐tolerant rodent brains and retina [31, 32, 33], and the PcG protein level is closely related with the outcome of IS [34]. Herein, we speculated that RNF2 may have a role to confer in IS injury via regulation of downstream protein ubiquitination.

In this study, we found that RNF2 was significantly upregulated in human postmortem brain tissue from patients with stroke and rat brain underwent middle cerebral artery occlusion (MCAO), which predominantly expressed in neurons and in the activated glial cells, such as microglia and astrocyte. RNF2 overexpression significantly decreased infarct size and attenuated neuron apoptosis induced by focal cerebral ischemia in rats. Consistently, RNF2 knockdown aggregated neuronal injury under ER stress inducer TM or OGD/R treatment. Mechanistically, RNF2 interacts directly with MANF in nuclear and promotes its monoubiquitination, thereby maintaining the stability of MANF, which confers protection against brain ischemia. Moreover, knockdown of MANF partly abolished the protective effect of RNF2 overexpression on TM‐induced neuron apoptosis, suggesting that RNF2 inhibits neuronal cell apoptosis dependently on MANF. Thus, RNF2 could be a potential therapeutic target for the treatment of IS.

## 2 Materials and Methods

### 2.1 Human Brain Tissue Samples

All paraffin‐embedded human postmortem brain tissue samples (three stroke and three non‐stroke patients) were obtained from the Anhui Provincial Key Laboratory for Brain Bank Construction and Resource Utilization. Informed consent for use of the human tissues for research was obtained in writing from all donors or the next of kin. All subjects were defined as normal controls or IS samples by pathologists. No subjects with prolonged agonal state were used; cause of death and related information are shown in Table S1. All the experiments related to human tissues were conducted in accordance with the Helsinki criteria and were also approved by the Ethics Committees of Anhui Medical University with the approval number 20200021.

### 2.2 Animals

Male Sprague–Dawley rats (6–8 weeks old, weight 150–200 g) were obtained from the Zhejiang Vital River Laboratory Animal Technology Company and kept under SPF conditions in the Experimental Animal Center of Anhui Medical University for 1 week prior to the experiment. Rats were housed in a temperature‐ and humidity‐controlled condition with a 12 h light/dark cycle. Standard laboratory food pellets and water were available ad libitum. All the experimental procedures for animal surgery were approved by the Animal Ethics Committee of Anhui Medical University with the approval number SCXK2019‐0001.

### 2.3 Transient Middle Cerebral Artery Occlusion Model

The focal cerebral ischemia rat model was performed as described previously [35]. Rats were anesthetized with 2% isoflurane mixed with air, and the body temperature was maintained using a homoeothermic blanket. Focal cerebral ischemia was induced by using MCAO with a suture [36]. Briefly, a nylon monofilament was inserted to the origin of the right MCA of rats. The filament was withdrawn at 2 h after occlusion to allow for reperfusion for 24 h. Sham‐operated rats underwent similar surgical procedures except for filament insertion. After the operation, mice were kept in warm cages for 2 h for recovery. The other details of materials and methods are presented in the Supporting Information.

## 3 Results

### 3.1 RNF2 Is Upregulated in the Brain After Focal Cerebral Ischemia

**FIGURE 1:**
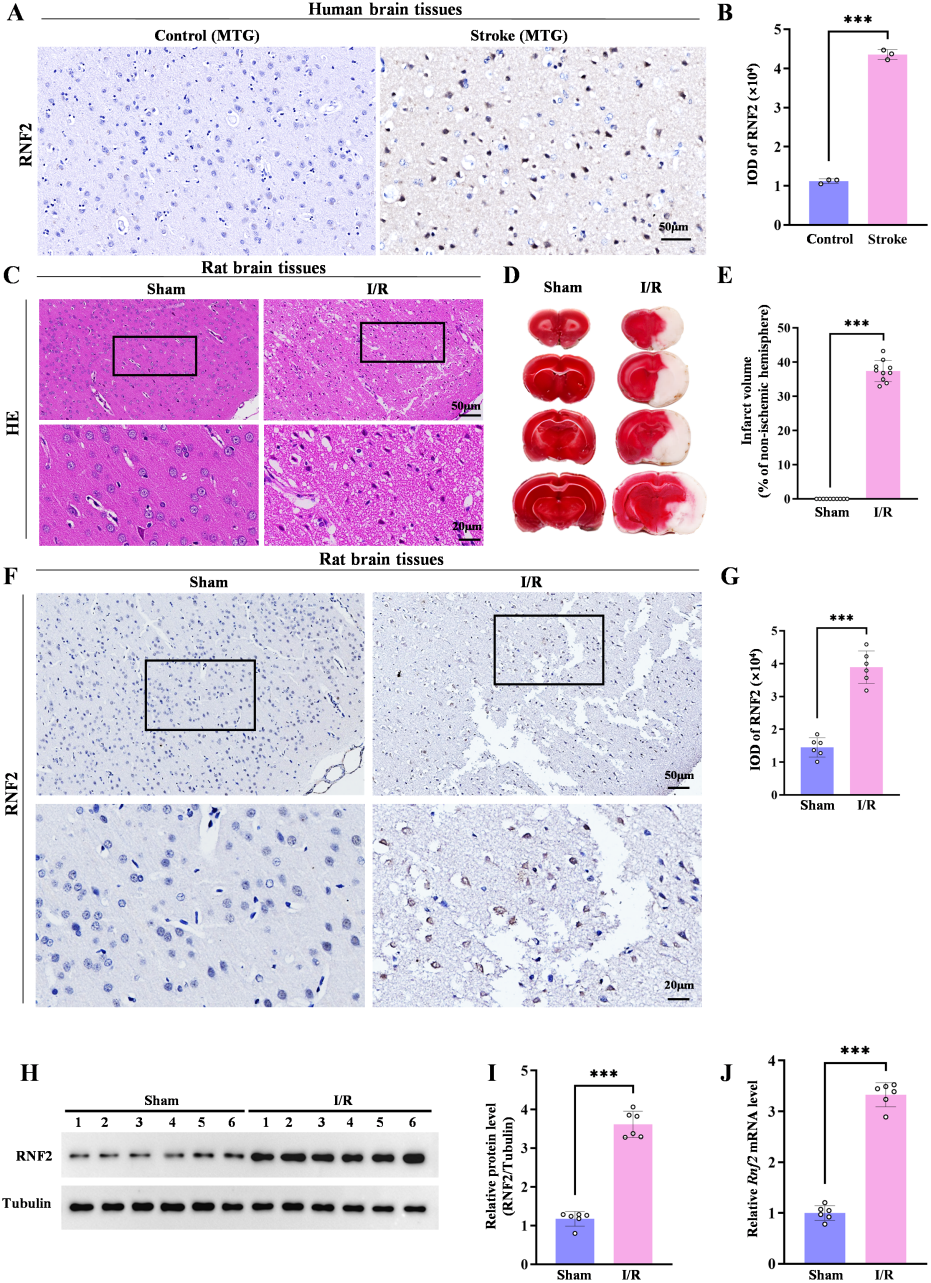
RNF2 is upregulated in stroke patients and the focal cerebral ischemic rat brain tissues. MCAO was performed for 2 h occlusion, followed by reperfusion for 24 h. (A) The RNF2‐positive cells were detected in MTG of stroke patients' brains by using immunohistochemistry with anti‐RNF2 antibody. MTG, middle temporal gyrus. Scale bar = 50 μm. (B) Quantitation of data as in A (*n* = 3; ***p < 0.001 vs. control; *t*‐test). (C) HE staining of the rat brain cortexes. (D) The first four slices of rat brain tissues were stained by TTC after MCAO, compared with sham. The normal brain tissue appeared red, while the infarction region showed white. (E) Quantitation of data as in A (*n* = 10; ***p < 0.001 vs. sham; *t*‐test). (F) The RNF2‐positive cells were detected in the rat cerebral cortex by using immunohistochemistry with anti‐RNF2 antibody. Upper panel scale bar = 50 μm, lower panel scale bar = 20 μm. (G) Quantitation of data as in F (*n* = 6; ***p < 0.001 vs. Sham; *t*‐test). (H, J) RNF2 protein (H) and mRNA (J) levels in I/R brain tissues were detected by western blot and qPCR, respectively. (I) Quantitation of data as in H (*n* = 6; **p < 0.01, ***p < 0.001 vs. sham; *t*‐test).

**FIGURE 2:**
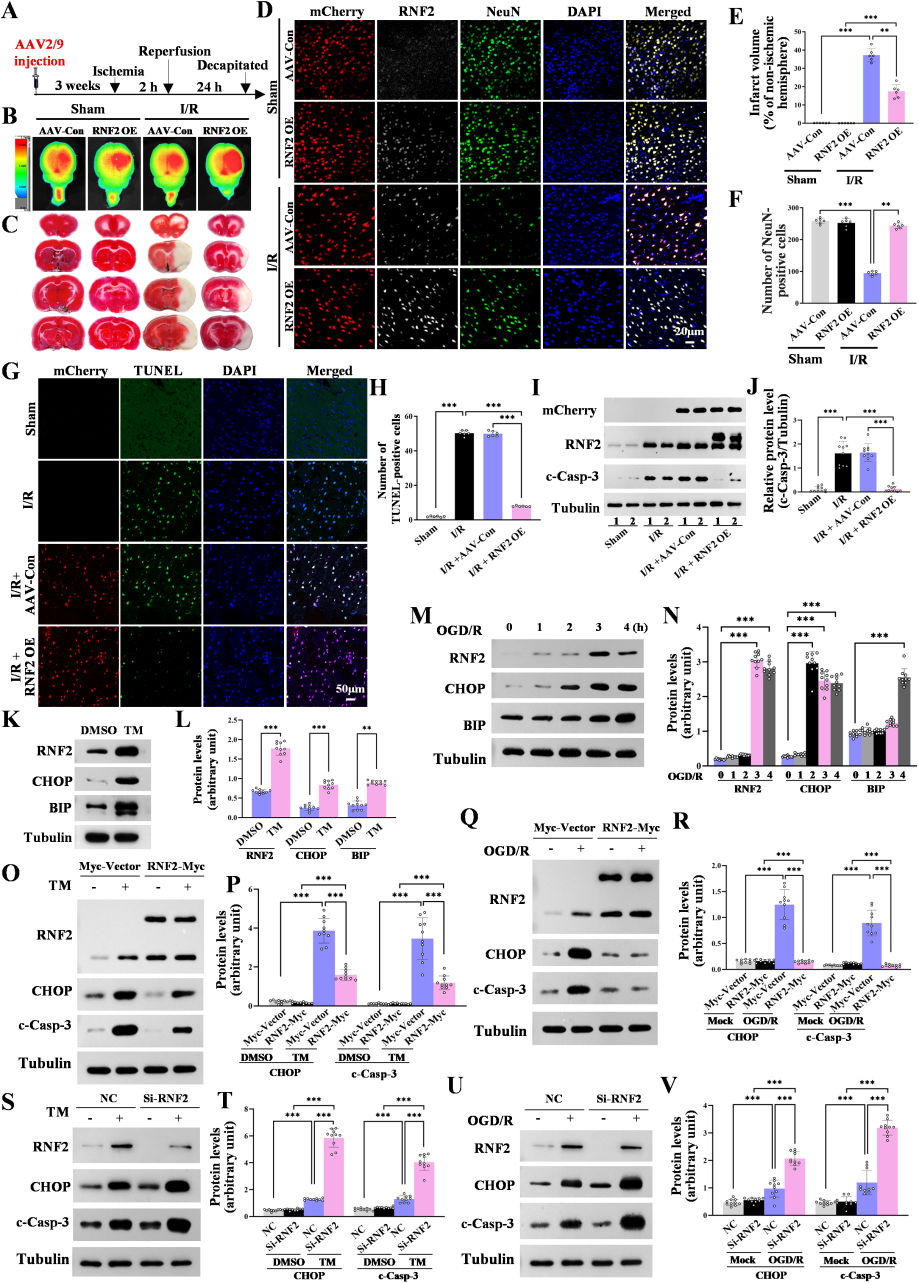
RNF2 overexpression attenuates I/R‐induced neuron loss and protects against TM and OGD/R‐induced nerve cells apoptosis. (A) Strategy for adeno‐associated viruses (AAV) administration. (B) Biodistribution of AAV. Fluorescence‐labeled AAV were stereotaxically injected into the lateral ventricle 3 weeks before I/R. At 24 h after I/R, the brain tissues with AAV‐Con or AAV‐RNF2 injection were harvested and fluorescence imaged by an in vivo imaging system. The gradation bar shows the relative levels of hexa‐peri‐hexabenzocoronene fluorescence intensity. (C) Photomicrograph of first four slices of brains treated with either AAV‐Con or AAV‐RNF2 before I/R. The brain tissues were stained by TTC at 24 h after I/R. The normal brain tissue appeared red, while the infarction region showed white. (D) RNF2 expression in the neurons of brain tissue. Brain samples were collected from the sham and ischemic rat brain tissue with AAV‐Con or AAV‐RNF2 injection 3 weeks before I/R. RNF2 expression in neurons was detected by double immunofluorescent staining for NeuN (white) and RNF2 (green) with anti‐NeuN and anti‐RNF2, respectively. mCherry is the virus's own fluorescence. Scale bar = 20 μm. (E, F) Quantitation of data as in C and D (*n* = 6; **p < 0.01, ***p < 0.001 vs. AAV‐Con; *t*‐test). (G) Apoptosis as detected by TUNEL assays rat brain tissues with AAV‐Con or AAV‐RNF2 injection 3 weeks before I/R. Scale bar = 50 μm. (H) Quantitation of data as in G (*n* = 6; ***p < 0.001; one‐way ANOVA followed by Tukey's test). (I) Representative western blot images of RNF2 expression with AAV‐Con or AAV‐RNF2 injection 3 weeks before I/R. c‐Casp‐3 protein level was detected using anti‐caspase 3 (active) antibody. (J) Quantitation of data as in I (*n* = 10; ***p < 0.001; one‐way ANOVA followed by Tukey's test). (K) TM treatment increases the expression of RNF2 in the N2a cells. N2a cells were treated with TM for 16 h. Western blot was used to detect the expression of RNF2. The proteins were detected with anti‐RNF2, anti‐CHOP/GADD153, and anti‐BIP antibody. Tubulin was used as a loading control. (L) Quantitation of data as in K (*n* = 10; **p < 0.01, ***p < 0.001 vs. DMSO; *t*‐test). (M) OGD/R treatment increases the expression of RNF2 in SH‐SY5Y cells. SH‐SY5Y cells were treated with OGD/R for 1, 2, 3, and 4 h, and then the cells were collected after 24 h of oxygen–glucose reoxygenation. Western blot was used to detect the level of RNF2. OGD/R: Oxygen–glucose deprivation/ reoxygenation. The proteins were detected with anti‐RNF2, anti‐CHOP/GADD153, and anti‐BIP antibody. Tubulin was used as a loading control. (N) Quantitation of data as in M (*n* = 10; ***p < 0.001 vs. 0 h; *t*‐test). (O, Q) RNF2 overexpression inhibits caspase‐3 activation and CHOP expression. N2a cells (O) or SH‐SY5Y (Q) cells were transiently transfected with the plasmids as indicated. The Myc‐Vector was used as the controls of Myc‐RNF2. After 36 h of transfection, the cells were treated with TM (2.5 μg/mL) for 16 h (O) or OGD for 3 h followed by reoxygenation for 24 h (Q). The proteins were detected with anti‐CHOP/GADD153 and anti‐cleaved caspases‐3 antibody. Tubulin was used as a loading control. (P, R) Quantitation of data as in O and Q (*n* = 10; ***p < 0.001 vs. Myc‐Vector; *t*‐test). (S, U) Knockdown of RNF2 aggravates caspase‐3 activation and CHOP expression. N2a cells (S) or SH‐SY5Y (U) cells were transiently transfected with the siRNA as indicated, and NC was used as the corresponding control. After 36 h of transfection, the cells were treated with TM (2.5 μg/mL) for 16 h (S) or OGD for 3 h followed by reoxygenation for 24 h (U). The proteins were detected with anti‐CHOP/GADD153 and anti‐cleaved caspases‐3 antibody, respectively. Tubulin was used as a loading control. (T, V) Quantitation of data as in S and U (*n* = 10; ***p < 0.001 vs. NC; *t*‐test).

Given that PcG proteins are increased in the ischemic‐tolerant rodent brain and overexpressing the PcG family proteins SCMH1 or BMI1 induces tolerance to ischemia, we asked whether RNF2 was involved in IS injury. We first analyzed the expression of RNF2 in human postmortem brain tissue samples with IS and rat brain under MCAO, respectively. RNF2 was widely expressed in the human ischemic brain compared to the control brain (Figure 1A,B). Consistently, the expression of RNF2 was also increased in the peri‐infarct area of I/R rats subjected to MCAO for 2 h followed by reperfusion for 24 h, which exhibits massive necrosis (Figure 1C,F,G) and infarction volume (Figure 1D,E). Furthermore, both protein and mRNA levels of RNF2 were upregulated in the ischemic brain, as revealed by quantitative polymerase chain reaction (qPCR) (Figure 1J), western blot analysis (Figure 1H,I), and relative qPCR (Figure S1A,B), implying a potential involvement of RNF2 in IS. To further verify if RNF2 was regulated by ischemic insult, we examined RNF2 expression in cultured neurons after OGD/R treatment and found that the RNF2 level was significantly increased, which was consistent with that in the MACO brain (Figure 2M,N).

**FIGURE 3:**
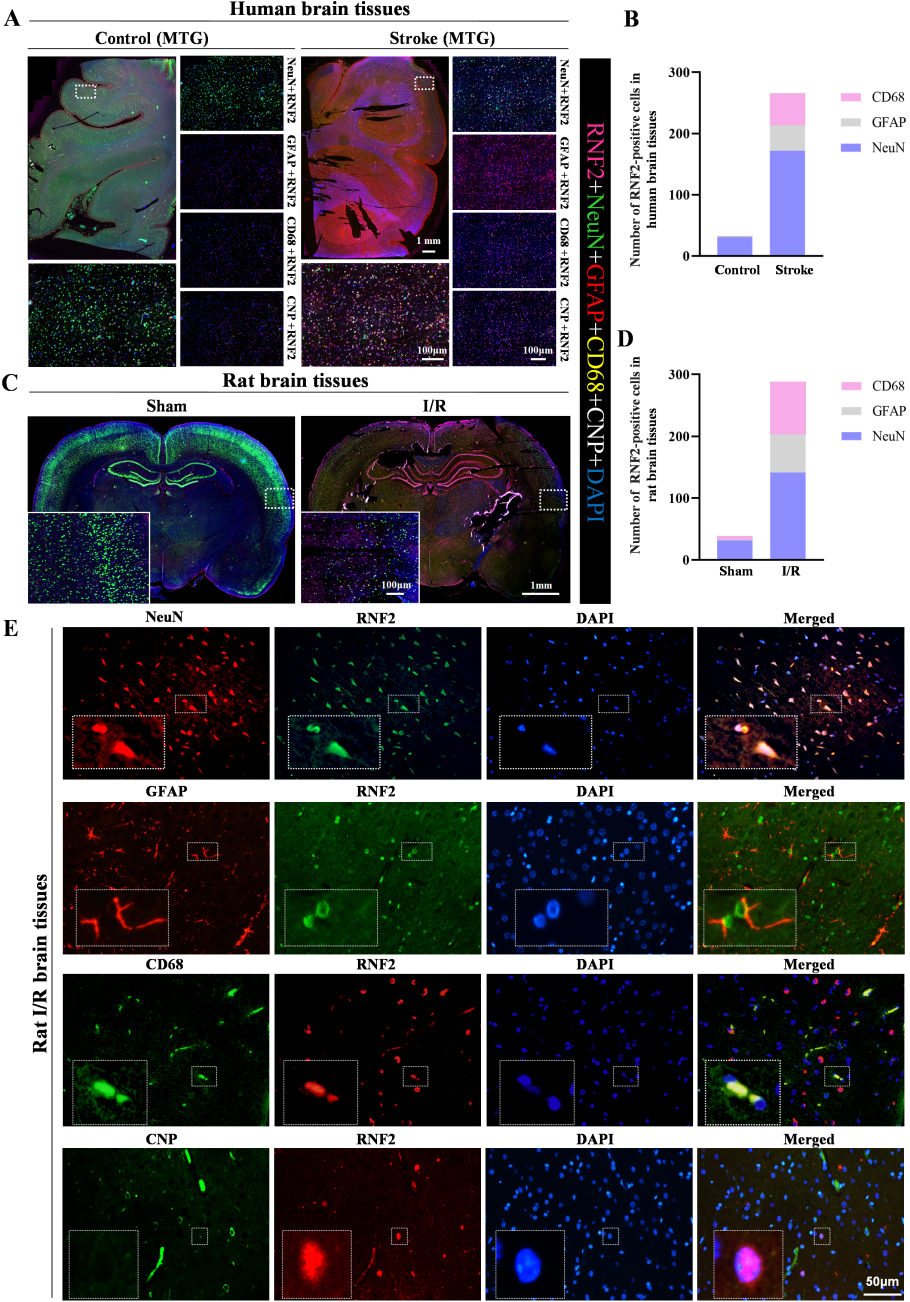
Expression of RNF2 in nerve cells. (A, C) Brain samples were collected from stroke and non‐stroke patients (A) and rat brain tissues (C). RNF2 expression in nerve cells was detected by pentuple‐fluorescence staining for RNF2 (rose red), NeuN (green), GFAP (red), CD68 (yellow), and CNP (white) with anti‐RNF2, anti‐NeuN, anti‐GFAP, anti‐CD68 and anti‐CNP, respectively. Whole tissue photo scale bar = 1 mm; small image scale bar = 100 μm. (B, D) Quantitation of data as in A and C. (E) Brain samples were collected from ischemic rat brain tissue 24 h after I/R. RNF2 expression in nerve cells was detected by double immunofluorescent staining. The top panel is double immunofluorescent staining for NeuN (red) and RNF2 (green) with anti‐NeuN and anti‐RNF2, respectively. The second panel is double immunofluorescent staining for glial fibrillary acidic protein (GFAP) (red) and RNF2 (green) with anti‐GFAP and anti‐RNF2, respectively. The third panel is double immunofluorescent staining for CD68 (green) and RNF2 (red) with anti‐CD68 and anti‐RNF2, respectively. The fourth panel is double immunofluorescent staining for CNP (green) and RNF2 (red) with anti‐CNP and anti‐RNF2, respectively. 2′3′‐cyclic nucleotide 3′‐phosphodiesterase, CNP. Scale bar = 50  μm.

Next, we examined the distribution of RNF2 in the human and rat brain. RNF2 was widely expressed in the brain with high levels under the ischemic condition but weakly expressed in the control brain. To investigate the cellular localization of RNF2 in the brain, we examined the expression of RNF2 in human and rat brains. Pentuple‐fluorescence immunofluorescent staining showed that most RNF2‐positive cells were NeuN‐positive neurons, while fewer RNF2‐positive cells were GFAP‐positive astrocytes or CD68‐positive microglia, but there were no CNP‐positive oligodendrocytes in I/R brain tissues (Figure 3A–D). Consistently, double‐labeled immunofluorescent staining showed that RNF2 can be differentially expressed in glial cells, including astrocytes and microglia, and that neurons are the major source of RNF2 in the rat MACO brain (Figure 3E). These results, together with our previous findings, indicate that I/R upregulates the expression of RNF2, especially in neurons.

### 3.2 RNF2 Overexpression Ameliorates Brain Injury Induced by MCAO

To explore the functional role of RNF2 in I/R injury, mCherry‐fluorescent‐labeled AAV2/9 virus carrying the RNF2 was stereotaxically injected into the unilateral lateral ventricle 3 weeks before MACO (Figure 2A and Figure S2A). Whole brain fluorescence images were collected using an in vivo imaging system at 3 weeks after AAV‐RNF2 or AAV‐Con injection, and fluorescent signals can be detected both in the left and in right hemispheres of the brain (Figure 2B and Figure S2A). Compared with that of AAV‐Con‐infected rats, RNF2 expression was acutely upregulated both in sham and I/R brains, indicating that RNF2 was successsfully transfected into brains (Figures S3A,B and S2B). In addition, most of mCherry‐positive cells are NeuN‐positive neurons, but also existed in some NeuN‐negative cells, suggesting that AAV‐RNF2 leads to a wide expression of RNF2 in the ischemia brain (Figure S2C). RNF2 overexpression markedly reduced the infarct volume after reperfusion compared with AAV‐Con‐injected rats (Figure 2C,E). Compared to the AAV‐Con‐injected I/R brain, the number of NeuN‐positive cells is remarkably increased in AAV‐RNF2‐injected rats following I/R induction, although there are no difference between AAV‐Con‐ and AAV‐RNF2‐transfected sham rat (Figure 2D,F), suggesting that RNF2 alleviates cerebral injury induced by IS.

### 3.3 RNF2 Inhibits I/R‐Induced Neuron Apoptosis and Protects Against TM and OGD/R‐Induced Nerve Cells Apoptosis

Given that RNF2 overexpression obviously increased NeuN‐positive cells after I/R injury, we wondered whether RNF2 facilitates neuroprotection against cerebral ischemia via inhibiting neuronal apoptosis. As we expected, RNF2 overexpression alleviated neuronal apoptosis in the cortex after MCAO, as shown by TUNEL staining (Figure 2G,H) and cleaved caspase‐3 (c‐casp‐3) (Figure 2I,J and Figure S4A–C), indicating a protective role of RNF2 in IS injury. Next, we used OGD/R and TM to mimic I/R in vitro and observed the effect of RNF2 overexpression or knockdown on neuronal apoptosis. TM treatment (2.5 μg/mL, 16 h) increased the expression of RNF2 in N2a cells, accompanied by upregulated levels of the ER stress marker BIP and pro‐apoptotic markers CHOP and c‐casp‐3 (Figure 2K,L,O,P). Consistently, glucose reoxygenation following OGD for 1, 2, 3, and 4 h gradually upregulated the levels of RNF2, BIP, CHOP, and c‐casp‐3 in SH‐SY5Y cells, especially at 3 h hypoxia and 24 h reoxygenation (Figure 2M,N,Q,R). RNF2 overexpression inhibited the activation of caspase‐3 and CHOP expression both in TM and OGD/R cells (Figure 2O–R), suggesting that RNF2 playing a protective role again I/R induced neuronal apoptosis. Then N2a cells were transiently transfected with RNF2‐siRNA and treated with TM for 16 h (Figure 2S,T). SH‐SY5Y cells were transiently transfected with RNF2‐siRNA and treated with OGD for 3 h and reoxygenated for 24 h (Figure 2U,V). The upregulated levels of c‐casp‐3 and CHOP in the RNF2 knockdown group suggested that RNF2 knockdown aggravates TM and OGD/R‐induced neuronal apoptosis. Additionally, RNF2 knockdown significantly increased the number of apoptotic cells detected by flow cytometry (Figure S5A,B). Together, these data show that RNF2 alleviates ischemia‐induced neuronal apoptosis.

### 3.4 RNF2 Interacts With MANF in the Nuclei

**FIGURE 4:**
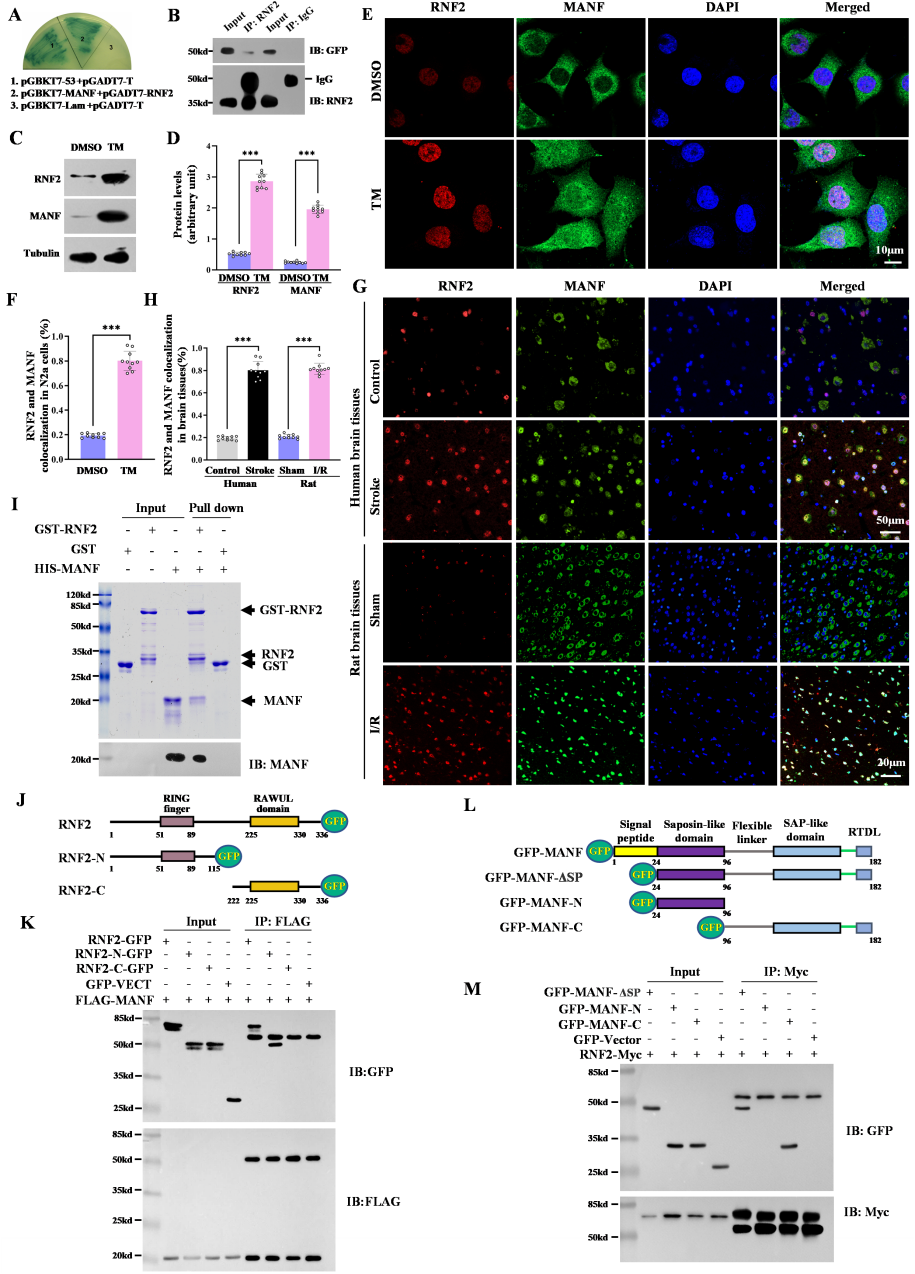
RNF2 interacts with MANF in the nuclei. (A) Interaction between RNF2 and MANF verified by the yeast two‐hybrid system. The yeast cells were co‐transformed and selected on supplemented minimal QDO/X plates. (1) Positive control pGBKT7‐53 and pGADT7T; (2) pGBKT7‐MANF and pGADT7‐RNF2; and (3) negative control pGBKT7‐Lam and pGADT7. (B) Interaction between RNF2 and MANF was verified by Co‐IP assay. N2a cells stably expressing GFP‐MANF were incubated with an anti‐RNF2 rabbit antibody immobilized on protein A/G beads (lane 2) and IgG antibody (lane 4) immobilized on protein A/G beads as negative control. The proteins were resolved by SDS‐PAGE, followed by western blotting analyses. Upper panel, GFP‐MANF was blotted with anti‐GFP; lower panel, RNF2 was blotted with anti‐RNF2. (C) TM‐induced expression of RNF2 and MANF. N2a cells were treated with TM (2.5 μg/mL) for 16 h. Cell lysates were analyzed by immunoblotting with the indicated antibodies. Tubulin was used as loading control. (D) Quantitation of data as in C (*n* = 10; ***p < 0.001 vs. DMSO; *t*‐test). (E) TM‐induced colocalization of RNF2 and MANF. Double labeling of RNF2 (red) and MANF (green) in N2a cells with TM (2.5 μg/mL) for 16 h. DAPI was used to counterstain the nuclei. Scale bar = 10 μm. (F) Quantitation of data as in E. Percentage of RNF2 and MANF localization cells in E (*n* = 10; ***p < 0.001 vs. DMSO; *t*‐test). (G) Colocalization of RNF2 and MANF in stroke patients and I/R brain tissues. Focal cerebral ischemia was performed using the method of right MCAO for 2 h followed by reperfusion for 24 h. Double‐labeled immunofluorescent staining was performed using antibodies against RNF2 (green) and MANF (red) in MTG of stroke patients and the ischemic cerebral cortex after MCAO. DAPI was used to counterstain the nuclei. The scale bar = 20 μm. MTG, middle temporal gyrus. Upper panel scale bar = 50 μm, lower panel scale bar = 20 μm. (H) Quantitation of data as in G. Percentage of RNF2 and MANF localization cells in G (*n* = 10; ***p < 0.001 vs. control or sham; *t*‐test). (I) His‐tagged human MANF was expressed in *Escherichia coli* BL‐21 and purified using Ni‐NTA agarose. Glutathione S‐transferase (GST) fusions were expressed in *Escherichia coli* BL‐21 and immobilized on glutathione‐Sepharose beads. GST‐RNF2 immobilized on glutathione‐Sepharose beads were incubated with recombinant His‐MANF and subjected to IB with anti‐MANF antibody. Ponceau staining of the PVDF membrane indicates expression of GST and GST‐RNF2. (J) Schematic diagram of RNF2 truncates. (K) Mapping the binding site of RNF2 and MANF by Co‐IP assay. The plasmids as indicated were co‐transfected to N2a cells. After 36 h transfection, the lysates were incubated with anti‐FLAG M2 affinity gel. After gel separation, the membrane was immunoblotted with antibodies against GFP and FLAG. (L) Schematic diagram of MANF truncates. (M) Mapping the binding site of RNF2 and MANF by Co‐IP assay. The plasmids as indicated were co‐transfected to N2a cells. After 36 h transfection, the lysates were incubated with an anti‐RNF2 rabbit antibody immobilized on protein A/G beads. The proteins were resolved by SDS‐PAGE followed by western blotting analyses. Upper panel, GFP‐MANF was blotted with anti‐GFP; lower panel, RNF2‐Myc was blotted with anti‐Myc.

To explore the mechanism underlying the neuroprotective effect of RNF2 in focal cerebral ischemia, we screened the RNF2‐interacting proteins using a yeast two‐hybrid method and found that MANF may be the candidate for RNF2. The interaction between MANF and RNF2 was verified by yeast back hybridization in which pGBKT7‐MANF and PGADT7‐RNF2 were co‐transformed into yeast gold strain. We found that the co‐transformed yeast grew normally and appeared blue on defective medium QDO/X, suggesting that RNF2 could interact with MANF in yeast (Figure 4A). Co‐IP also confirmed the interaction between MANF and RNF2 in vivo (Figure 4B). As we mentioned before, ER stress inducer TM obviously upregulated MANF and RNF2 expression (Figure 4C,D). MANF is mainly located in the cytoplasm in normal cells, while TM elicits MANF translocation to the nucleus, which is colocalized with RNF2 in the N2a cell (Figure 4E,F). Consistently, RNF2 levels in the nucleus were increased in the brain tissue from stroke patients and I/R rats and co‐existed with MANF under ischemic injury (Figure 4G,H). GST pull‐down assay further confirm the direct interaction between MANF and RNF2 (Figure 4I). To further clarify the functional interacting domain between RNF2 and MANF, the plasmids carrying different RNF2 or MANF truncation mutants were transfected into N2a cells, following co‐IP experiments. As shown in Figure 4I,K, the RNF2 mutant lacking the RING‐finger domain failed to interact with MANF, while the MANF mutant lacking the C‐terminal including SAP‐like domain and RTDL sequence failed to interact with RNF2, indicating that the C‐terminal of MANF was able to bind to the N‐terminal of RNF2 (Figure 4J–M).

### 3.5 RNF2 Regulates the Stability of MANF by Monoubiquitylation

**FIGURE 5:**
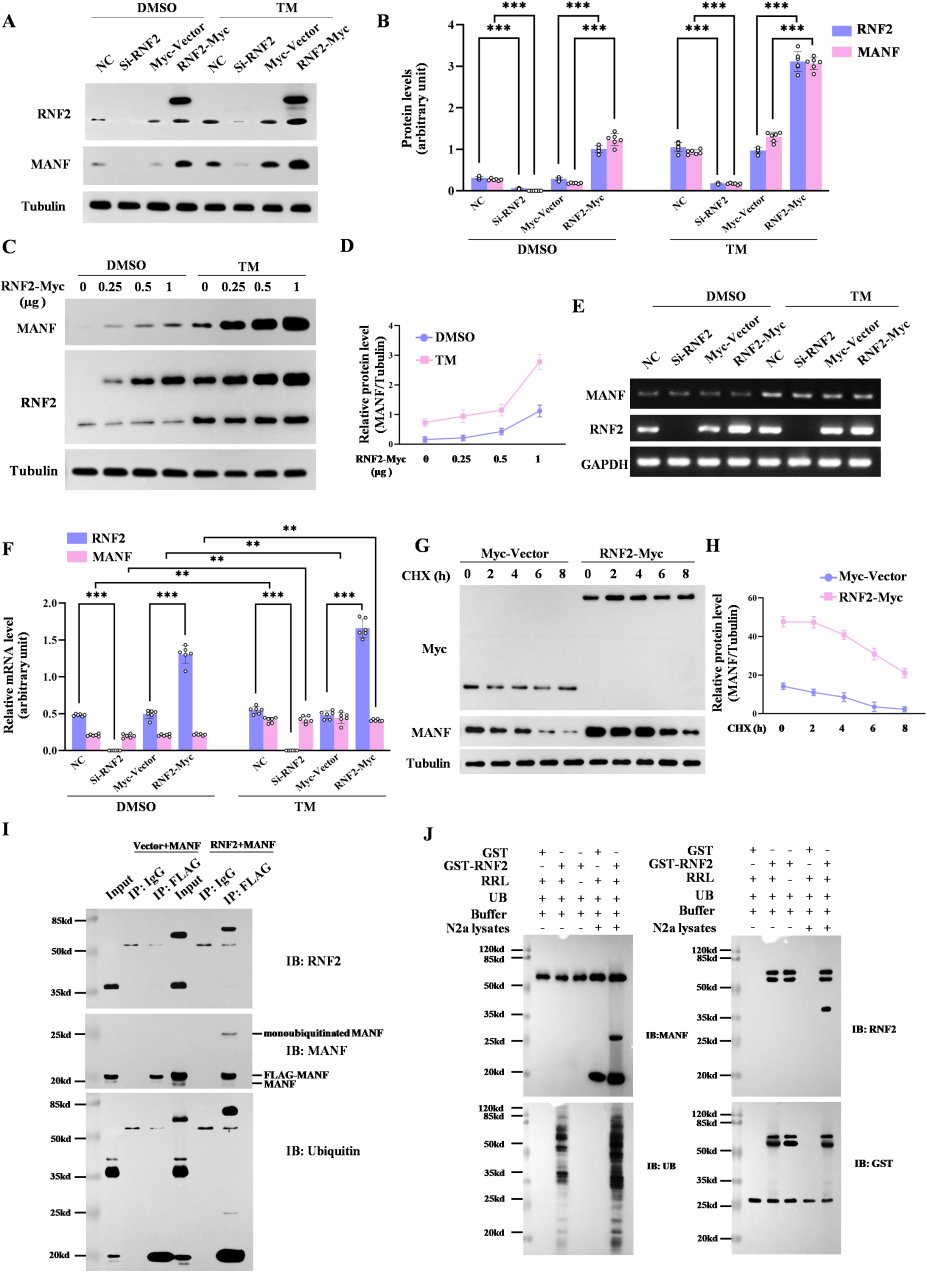
RNF2 regulates MANF stability and mediates MANF monoubiquitylation in vivo and in vitro. (A) RNF2 regulates the MANF protein level. N2a cells were transfected with RNF2‐siRNA or RNF2‐Myc and treated with TM (2.5 μg/mL) for 16 h at 36 h after transfection. DMSO was used as control. The protein levels of RNF2 and MANF were detected with anti‐RNF2 and anti‐MANF antibodies, respectively. Tubulin was used as a loading control. (B) Quantitation of data as in A (*n* = 6; ***p < 0.001 vs. NC or Myc‐Vector; *t*‐test). (C) RNF2 upregulates MANF in a dose‐dependent manner. N2a cells were transiently transfected with RNF2‐Myc plasmid (0, 0.25, 0.5, 1.0 μg). After 36 h of transfection, the cells were treated with TM for 16 h. The levels of MANF and RNF2 were detected by western blot assay. Tubulin was used as a loading control. (D) Variation curve of the MANF level in C. (E) RNF2 had no effect on the MANF transcription level. N2a cells were transfected with RNF2‐siRNA or RNF2‐Myc for 36 h. The expressions of RNF2 and MANF in N2a cells were detected by using relative quantitative RT‐PCR assay. Tubulin was used as loading control. (F) Quantitation of data as in E (*n* = 6; *p < 0.05, **p < 0.01, ***p < 0.001 vs. NC or Myc‐Vector; *t*‐test). (G) Effect of treatment of cycloheximide (CHX; 20 μg/mL) on the levels of MANF protein in N2a cells after transfection with Myc‐Vector or RNF2‐Myc. (H) Variation curve of the MANF level in the G. (I) Monoubiquitination of MANF by RNF2. N2a cells were co‐transfected with GFP‐RNF2 and FLAG‐MANF plasmids and treated with 2.5 μg/mL TM for 16 h. MANF was immunoprecipitated with anti‐FLAG. The bound proteins were detected by western blot assay. (J) Protein G‐Sepharose‐bound MANF protein was immunopurified from N2a cell lysates. The immunoprecipiates were subjected to in vitro ubiquitination reactions for 2 h through use of RRL and recombinant GST‐tagged human RNF2. Then the products of the in vitro ubiquitination assay were eluted with SDS‐sample buffer, resolved by 8% SDS‐PAGE, and immunoblotted with anti‐MANF (left top panel), anti‐UB (left bottom panel), anti‐RNF2 (right top panel), anti‐GST (right bottom panel) antibodies, respectively. UB, ubiquitin.

Since RNF2 is a E3 ubiquitin ligase, we suspected that MANF might be a substrate of RNF2. First, we investigated the mRNA and protein levels of MANF in N2a cells transfected with RNF2‐overexpressing plasmids or siRNA, respectively, along with TM treatment. Obviously, the protein levels of MANF increased in RNF2 overexpressing cells and decreased in RNF2 knockdown cells (Figure 5A,B). Further study showed that RNF2 could dose‐dependently upregulate MANF protein levels with or without TM treatment (Figure 5C,D). However, we found that the altered expression of RNF2 did not affect the mRNA levels of MANF (Figure 5E,F), suggesting that RNF2 regulation of the MANF level merely occurs at the protein level. To further assess the effect of RNF2 on MANF protein stability, we inhibited de novo protein synthesis by cyclohexamide (CHX) and measured MANF protein levels in the RNF2‐overexpressed cells. Consistently, RNF2 significantly enlonged the half‐life of MANF in N2a cells treated with CHX (Figure 5G,H), which indicated that RNF2 stabilized MANF protein expression.

Next, we assessed whether E3 ubiquitin ligase RNF2 could mediate the ubiquitination of MANF, which makes it more stabilized. Co‐IP results showed that monoubiquitinated MANF was markedly increased in the presence of RNF2 overexpression (Figure 5I). The in vitro ubiquitination experiment further verifies that RNF2 facilitates MANF monobiquitination (Figure 5J). Together, these data show that RNF2 mediated MANF monoubiquitination to stabilize MANF.

### 3.6 RNF2 Enhances MANF Nuclear Translocation Under ER the Stress Condition

**FIGURE 6:**
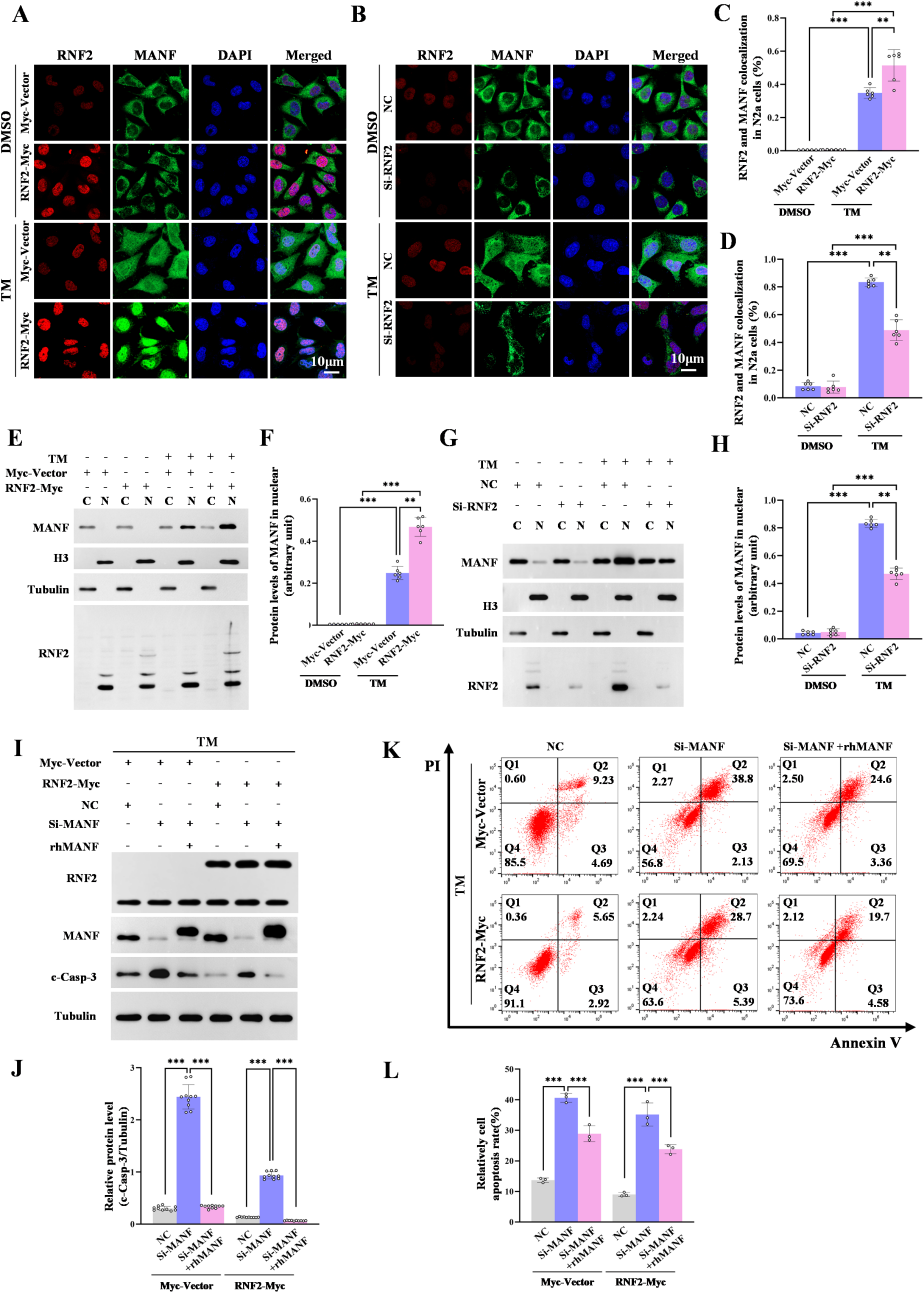
RNF2 enhances MANF nuclear transformation and inhibits neuronal cells apoptosis dependently on MANF. RNF2 interacts with MANF in N2a cells. N2a cells were transfected with RNF2‐Myc or RNF2‐siRNA and treated with TM (2.5 μg/mL) for 16 h at 36 h after transfection. (A, B) RNF2 (red) and MANF (green) were detected by immunofluoscent staining in N2a cells. DAPI was used to counterstain the nuclei. The scale bar = 10 μm. (C, D) Quantitation of data as in A and B. Percentage of RNF2 and MANF colocalization cells in A and B. (*n* = 6; **p < 0.01, ***p < 0.001 vs. Myc‐Vector or NC; *t*‐test). (E, G) RNF2 enhances MANF nuclear transformation. N2a cells were transfected with RNF2‐Myc (E) or RNF2‐siRNA (G), or corresponding controls and treated with or without TM for 16 h at 36 h posttransfection. The cytoplasmic and nuclear proteins were extracted and western blotting was performed. Tubulin and histone H3 were used as cytoplasmic and nuclear markers, respectively. C, cytoplasm; N, nucleus. (F, H) Quantitation of data as in E and G (*n* = 6; **p < 0.01, ***p < 0.001 vs. Myc‐Vector or NC; *t*‐test). RNF2‐Myc plasmid was transfected into MANF knockdown N2a cells, or corresponding controls and treated with TM for 16 h at 36 h posttransfection. N2a cells were treated with rhMANF for 2 h before harvesting. (I) Cleaved caspase‐3 expression was detected using Western blot. (J) Quantitation of data as in I (*n* = 10; ***p < 0.001; one‐way ANOVA followed by Tukey's test). (K) Flow cytometry showing apoptotic N2a cells. (L) Quantitation of data as in K (*n* = 3; **p < 0.01, ***p < 0.001; one‐way ANOVA followed by Tukey's test).

Given the functions of RNF2 in promoting the stabilization of MANF via monoubiquitination, we inferred that RNF2 might facilitate MANF nuclear translocation under stroke stress. We found that MANF mostly expressed in the cytoplasm in control but translocated into nuclear and colocalized with RNF2 upon TM stimulation. Moreover, RNF2 overexpression further increased the expression of MANF in the nucleus and facilitated the colocalization between RNF2 and MANF in the presence of TM (Figure 6A,C). To further verify the effect of RNF2 on MANF nuclear translocation, total cellular and nuclear proteins were extracted with nuclear plasma separation kit and western blot result showed that RNF2 enhanced nuclear MANF levels under TM treatment (Figure 6E,F). Consistent with this result, RNF2 knockdown attenuated TM‐induced MANF nuclear translocation (Figure 6B,D,G,H).

Mechanistically, RNF2 stabilized MANF and facilitated MANF nuclear translocation under the ER stress condition.

### 3.7 RNF2 Inhibits Neuron Apoptosis Dependently on MANF

To investigate whether RNF2 functions dependently on MANF in the ischemic brain, c‐casp‐3 expression was detected in MANF knockdown cells. We found that MANF siRNA obviously enhanced c‐casp‐3 levels, but the effect was weakened to some extent in RNF2 overexpressing cells (Figure 6I,J, Lane 5 vs. Lane 2). rhMANF was proved to play a neuroprotective role in a series of brain injury, although the underlying mechanism is not very clear. Here, we observed that rhMANF treatment decreased caspase‐3 activation whether RNF2 overexpressing or not, which is consistent with our previous studies (Figure 6I,J, Lane 3 vs. Lane 2, Lane 6 vs. Lane 5). However, in RNF2 overexpressing cells, c‐casp‐3 levels were more significantly relieved accompanied by rhMANF treatment (Figure 6I,J, Lane 6 vs. Lane 3). Flow cytometry analysis (Figure 6K,L) and TUNEL staining (Figure S6A,B) further proved that the inhibitory effect of RNF2 on neuron apoptosis might be abolished partially by MANF knockdown and reversed by rhMANF. These findings suggest that RNF2 inhibits neuronal cells apoptosis partly dependently on MANF.

## 4 Discussion

In this study, we provided evidences for the first time that RNF2 was significantly upregulated in human and rat ischemia brain and RNF2 overexpression alleviated brain injury induced by MCAO. We further proved that RNF2 could directly interact with MANF in nucleus, induce MANF monoubiquitination and stabilization, and finally promote neuronal survival.

MANF has been originally described as a neurotrophic factor but has subsequently been found to be an important UPR‐responsive gene with an active role in maintaining ER homeostasis [37, 38]. MANF has been proved to have a protective effect on the IS outcome [11, 39]. The protein expression of neuronal MANF was found elevated 2–48 h post‐stroke after transient MCAO in the peri‐infarct region [36, 40]. When exogenous MANF is administered into the brain before or a few hours after stroke, it has neuroprotective effects on IS models [11, 41], and the CxxC‐motif is indispensable for the neuroprotective effect of MANF in IS, possibly because of its importance in maintaining MANF's structural conformation [41]. It is also reported that ER stress and inflammation caused MANF to relocalize to the nuclei, consequently interacted with the DNA binding domain of p65 through its C‐terminal SAP‐like domain, and inhibited p65 activation in several in autoimmune diseases and inflammatory diseases, which suggest that MANF may be a negative regulator of inflammation [15, 42]. It is well known that neuroinflammation is a key element underlying the cerebral injury caused by IS. Therefore, we suspected that the neuroprotective effect of RNF2 might be related to the anti‐inflammatory function of MANF.

RNF2 is a member of the PcG family of proteins, which has been recognized to play crucial roles in cancer development. This is the first report to demonstrate that RNF2 ameliorated cerebral IS injury. Although there was a small amount of RNF2 expression in normal brain tissue, the RNF2‐positive cells were neurons, not glia in the human ischemic brain. However, severe cerebral ischemia could induce RNF2 expression in glial cells, including microglia and astrocytes, but the neurons are still a major source of RNF2, which is similar to the characteristic of MANF expression in the ischemic rat brain. Collectively, these results indicate that both RNF2 and MANF can be induced by ischemic injury and differentially expressed in glial cells, although the role of RNF2 and MANF in neuron and glial cells is not clear and deserved further study.

In this study, RNF2 overexpression by lateral ventricular injection of AAV‐RNF2 significantly reduced the volume of cerebral infarction and improved the neuron survival of cerebral ischemic rats. In contrast, RNF2 knockdown aggregated neuron apoptosis in either ER stress or OGD/R condition, suggesting that RNF2 confers protection against brain ischemia. However, the mechanism is still obscure. Interestingly, we found that the protein expression of MANF showed a consistent trend with that of the RNF2, but its transcription level was not affected by RNF2. Thus, we speculated that RNF2 played a protective role in IS by stabilizing MANF protein levels.

Some transcription factors, such as AP1 and XBP1s, were reported to regulate the transcription of MANF. However, how MANF is regulated in post‐translation modification remains largely unknown. Ubiquitination is one of the most important post‐translational modification that controls the stability, function, and activity of proteins. In our study, RNF2 increased MANF monoubiqutination, suggesting that RNF2 is an E3 for MANF protein. The specificity of the ubiquitin conjugation system is provided by E3 ligase through its direct interaction with substrates. Structurally MANF consists of N‐terminal Saposin‐like domain and the C‐terminal SAP‐like domain that are connected by a flexible linker region ([43, 44]; Figure 4L). The N‐terminal domain of MANF has been shown to bind sulfatides (3‐O‐sulfogalactosylceramide) that are enriched in myelin [45, 46]. The structure of the C‐terminal domain of MANF was strikingly similar to the Ku70 C‐terminal SAP‐like domain, which is related to its anti‐apoptotic effect [44]. It should be noted that the protective activity of MANF in vitro was abolished with the deletion of the C‐terminal RTDL sequence, but the protein was biologically active and exhibited protective effects on IS in vivo. Thus, only the C‐terminal CxxC (149–152 aa) is essential for the protective activity of MANF in IS in vivo and antagonizes cell death [41, 47]. In the present study, we demonstrated that C‐terminal of MANF, including the flexible linker, SAP‐like domain, and RTDL, is responsible for interacting with RNF2.

RNF2 consists of three parts, a well‐folded fragment N‐terminal domain [48], C‐terminal domain [49, 50], and flexible linker [51]. PcG targeting to different chromatin locations relies, in part, on binding partners of C‐RING1B that are diverse in sequence and structure [50]. RING finger motif and flanking sequences form an interacting platform for PcG and non‐PcG proteins [48]. They often form homo‐ or heterodimeric pairs [52, 53], as in the Bmi1‐Ring1B complex, where each protein contains an N‐terminal RING domain through which the proteins heterodimerize to form a functional E3 ligase [48]. According to our study, the N‐terminal domain of RNF2 contributes to its E3 activity and interaction with MANF.

As we know, target proteins can be modified through mono‐ or multi‐ubiquitination at different lysine sites by ubiquitin. In general, the N‐terminal lysine is more susceptible to being polyubiquitinated, while the C‐terminal lysine is more susceptible to being monoubiquitinated. There are 22 lysine residues in MANF; importantly, there are 16 lysine residues in the C‐terminal [54], which may be the reason why MANF can be monoubiquitinated by RNF2. However, the specific ubiquitination site of MANF warrants further investigation. Furthermore, MANF knockdown effectively abolished the protective effect of RNF2 overexpression after cerebral ischemia. Therefore, these results suggested that RNF2 functions at least partially dependent on MANF.

In our study, we found that RNF2 mediates MANF monoubiquitylation and enhances MANF nuclear translocation in vivo and in vitro. As we know, monoubiquitination plays roles in proteasome‐unrelated events, such as protein trafficking and interaction with other proteins [55, 56]; the monoubiquitination of MANF might be involved in nuclear transport. In future studies, we will further investigate these issues.

## 5 Conclusion

Our study demonstrated that RNF2 was strongly upregulated in the ischemic brain and alleviated cerebral ischemic injury possibly by mediating MANF monoubiquitination and stabilization, resulting in enhanced nucleus translocation of MANF, which probably provides neuroprotection against neuronal apoptosis. Therefore, RNF2 could be a novel therapeutic target for the intervention of IS.

## Author Contributions

Yuxian Shen and Lijie Feng designed the study, supervised the experiments, and revised the manuscript draft. Yujun Shen performed the experiments, analyzed the data, and wrote the manuscript. Jinfeng Wang, Junxing Liang, and Ying Chen performed the experiments. Xueyan Wu participated in preparing patient samples. Zhenhua Ren and Jiangning Zhou conducted the pentuple‐fluorescence staining experiment and analyzed the data.

## Ethics Statement

All the experiments related to human tissues were conducted in accordance with the Helsinki criteria and were also approved by the Ethics Committees of Anhui Medical University with the approval number 20200021. All the experimental procedures for animal surgery were approved by the Animal Ethics Committee of Anhui Medical University with the approval number SCXK2019‐0001.

## Consent

Complete written informed consent has been obtained from the involved patients or from the parent, guardian, or power of attorney of the involved patients; they have given approval for the publication of this study and accompanying images.

## Conflicts of Interest

The authors declare no conflicts of interest.

## Supporting information


**Data S1.** Supporting Information.

**Figure S1.** RNF2 mRNA levels are upregulated in the focal cerebral ischemic rat brain tissues. MCAO was performed for 2 h occlusion followed by reperfusion for 24 h. (A) RNF2 mRNA levels in I/R brain tissues were detected by reverse transcription PCR. (B) Quantitation of data as in A (*n* = 6; ***p < 0.001 vs. sham; *t*‐test).

**Figure S2.** Intracerebral ventricular injection of adeno‐associated virus to overexpressing RNF2 in the ischemic cortex of rats. (A) Three weeks before MCAO, AAV were stereotaxically injected into the right lateral ventricle as follows: A/P = −1.6 mm, L/M = +1.5 mm, D/V = −4.5 mm. AAV successfully infected rat brain tissue and successfully expressed mCherry protein in brain tissue. Scale bar = 1500 μm. (B) Brain samples were collected from the sham and ischemic rat brain tissue with AAV‐RNF2 injection 3 weeks before I/R. The red fluorescent protein mCherry was expressed in the rat cortex, indicating that AAV‐RNF2 can express RNF2 protein in the ischemic cortex. Scale bar = 50 μm. (C) Immunofluorescence staining detected that AAV can infect neurons. NeuN‐positive cells were detected by immunofluorescence staining with the anti‐NeuN antibody. mCherry is the virus’s own fluorescence. Scale bar = 20 μm.

**Figure S3.** RNF2 expression was acutely upregulated in the AAV‐RNF2‐infected rat brain. AAV were stereotaxically injected into the lateral ventricle 3 weeks before I/R; AAV are successfully transfected into nerve cells and effectively alter the expression of RNF2. (A) RNF2 expression was detected by immunohistochemistry. Upper panel scale bar = 50 μm, lower panel scale bar = 20 μm. (B) Quantitation of data as in A. (*n* = 6; **p < 0.01, ***p < 0.001 vs. AAV‐Con; *t*‐test).

**Figure S4.** AAV‐mediated RNF2 overexpression inhibits I/R‐induced neuron apoptosis. AAV were stereotaxically injected into the lateral ventricle 3 weeks before I/R; AAV are successfully transfected into nerve cells and effectively alter the expression of RNF2. (A) Stably expressing RNF2 with the mCherry tag inhibits caspase‐3 activation detected by immumohistochemical staining and immunofluorescent staining using antibody against c‐Casp‐3 (green) in the ischemic cerebral cortex. Scale bar = 50 μm. (B, C) Quantitation of data as in A. (*n* = 6; **p < 0.01, ***p < 0.001; one‐way ANOVA followed by Tukey’s test).

**Figure S5.** RNF2 protects against TM‐induced nerve cells apoptosis. N2a cells were transiently transfected with the plasmids and siRNA as indicated. After 36 h of transfection, the cells were treated with TM (2.5 μg/mL) for 16 h. (A) Flow cytometry showing apoptotic N2a cells. (B) Quantitation of data as in A. (*n* = 3; *p < 0.05, ***p < 0.001 vs. Myc‐Vector or NC; *t*‐test).

**Figure S6.** RNF2 inhibits neuronal cells apoptosis dependently on MANF. RNF2‐Myc plasmid was transfected into MANF knockdown N2a cells, or corresponding controls and treated with TM for 16 h at 36 h posttransfection. N2a cells were treated with rhMANF for 2 h before harvesting. (A) Apoptosis as detected by TUNEL assays. Scale bar = 20 μm. Magnified photo scale bar = 10 μm. (B) Quantitation of data as in A (*n* = 3; **p < 0.01, ***p < 0.001; one‐way ANOVA followed by Tukey’s test).

**Figure S7.** AAV‐mediated RNF2 overexpression improves the Zea Longa scores. (A) The time–effect relationship of AAV‐mediated RNF2 overexpression on the Zea Longa score. AAV‐Con or AAV‐RNF2 were stereotaxically injected into the lateral ventricle 3 weeks before I/R. The Zea Longa score was evaluated at 24 h and on the days 2, 4, 6, 8, 10, 12, and 14 after I/R. Data are presented as the means ± SEM. (*n* = 8; *p < 0.05 vs. AAV‐Con; *t*‐test). (B) Zea Longa score was evaluated at 24 h after I/R. Data are presented as the means ± SEM. (*n* = 10; *p < 0.05, ****p < 0.0001; one‐way ANOVA followed by Tukey’s test). (C) Zea Longa score was evaluated at 4 days after I/R. Data are presented as the means ± SEM. (*n* = 5; *p < 0.05, ***p < 0.001; one‐way ANOVA followed by Tukey’s test). (D) Zea Longa score was evaluated at 10 days after I/R. Data are presented as the means ± SEM. (*n* = 4; *p < 0.05, ***p < 0.001; one‐way ANOVA followed by Tukey’s test). (E) Zea Longa score was evaluated at 14 days after I/R. Data are presented as the means ± SEM. (*n* = 4; *p < 0.05, **p < 0.01; one‐way ANOVA followed by Tukey’s test).

**Figure S8.** OGD/R treatment increases the expression of RNF2 and MANF in SH‐SY5Y cells. (A) SH‐SY5Y cells were treated with OGD/R for 3 h, and the cells were collected after 24 h of oxygen–glucose reoxygenation. Western blot was used to detect the level of RNF2 and MANF. (B) Quantitation of data as in A (*n* = 3; ***p < 0.001 vs. Mock; *t*‐test). (C) Repeat three dishes of cells as in A. (D) Quantitation of data as in C (*n* = 3; ***p < 0.001 vs. Mock; *t*‐test).

**Figure S9.** RNF2 regulates the MANF protein level but not the transcription level in SH‐SY5Y cells with OGD/R treatment. SH‐SY5Y cells were transfected with RNF2‐siRNA or RNF2‐Myc and treated with OGD/R at 36 h after transfection. Mock means without OGD/R treatment. The protein levels of RNF2 and MANF were detected with anti‐RNF2 and anti‐MANF antibodies, respectively. Tubulin was used as a loading control. (B) Quantitation of data as in A (*n* = 4; ***p < 0.001 vs. NC or Myc‐Vector; *t*‐test). (C) RNF2 upregulates MANF in a dose‐dependent manner. SH‐SY5Y cells were transiently transfected with RNF2‐Myc plasmid (0, 0.25 , 0.5, 1.0 μg). After 36 h of transfection, the cells were treated with OGD/R. The levels of MANF and RNF2 were detected by western blot assay. Tubulin was used as a loading control. (D) Variation curve of the MANF level in C. (E) RNF2 had no effect on the MANF transcription level. SH‐SY5Y cells were transfected with RNF2‐siRNA or RNF2‐Myc for 36 h. The expressions of RNF2 and MANF in SH‐SY5Y cells were detected by using relative quantitative RT‐PCR assay. GAPDH was used as loading control. (F) Quantitation of data as in E (*n* = 3; **p < 0.01, ***p < 0.001 vs. NC or Myc‐Vector; *t*‐test).

**Figure S10.** RNF2 interacts with MANF in SH‐SY5Y cells. SH‐SY5Y cells were transfected with RNF2‐Myc or RNF2‐siRNA and treated with OGD/R at 36 h after transfection. (A, C) RNF2 (red) and MANF (green) were detected by immunofluoscent staining in SH‐SY5Y cells. DAPI was used to counterstain the nuclei. The scale bar = 10 μm. (B, D) Quantitation of data as in A and C. Percentage of RNF2 and MANF colocalization cells in A and B. (*n* = 6; **p < 0.01, ***p < 0.001, ***p < 0.0001 vs. Myc‐Vector or NC; *t*‐test).

**Figure S11.** RNF2 inhibits neuronal cells apoptosis dependently on MANF. SH‐SY5Y cells were transfected with RNF2‐Myc or RNF2‐siRNA and treated with OGD/R at 36 h after transfection. (A) The proteins were detected with anti‐bcl‐2, anti‐bax and anti‐cleaved caspases‐3 antibody, respectively. Tubulin was used as a loading control. (B) Quantitation of data as in A (*n* = 3; **p < 0.01, ***p < 0.001 vs. NC or Myc‐Vector; *t*‐test). (C) Bax/Bcl‐2 ratio was evaluated as in A (*n* = 3; **p < 0.01, ***p < 0.001 vs. NC or Myc‐Vector; *t*‐test). (D) RNF2 overexpression protects against OGD/R‐induced nerve cells apoptosis. SH‐SY5Y cells were transiently transfected with the plasmids and siRNA as indicated. After 36 h of transfection, the cells were treated with OGD/R. (D) Flow cytometry showing apoptotic SH‐SY5Y cells. (E) Quantitation of data as in D (*n* = 3; ****p < 0.0001 vs. Myc‐Vector or NC; *t*‐test).

**Figure S12.** RNF2 inhibits neuronal cells apoptosis dependently on MANF. RNF2‐Myc plasmid was transfected into MANF knockdown SH‐SY5Y cells, or corresponding controls and treated with OGD/R at 36 h posttransfection. SH‐SY5Y cells were treated with rhMANF for 2 h before harvesting. (A) Flow cytometry showing apoptotic SH‐SY5Y cells. (B) Quantitation of data as in A (*n* = 3; *p < 0.05, ***p < 0.001, ****p < 0.0001; one‐way ANOVA followed by Tukey’s test). (C) Apoptosis as detected by TUNEL assays. Scale bar = 20 μm. Magnified photo scale bar = 10 μm. (D) Quantitation of data as in C (*n* = 3; **p < 0.01, ***p < 0.001; one‐way ANOVA followed by Tukey’s test).

## Data Availability

Research data are not shared.
