# Supplementary material for: E3 Ubiquitin Ligase Ring Finger Protein 2 Alleviates Cerebral Ischemia–Reperfusion Injury by Stabilizing Mesencephalic Astrocyte‐Derived Neurotrophic Factor Through Monoubiquitination
Source: CNS Neurosci Ther. 2024 Nov 30;30(11):e70136. doi: 10.1111/cns.70136 (PMC11607471; doi:10.1111/cns.70136)
Supplement: Supplementary file 1 — Data S1. Supporting Information. [file CNS-30-e70136-s003.docx]

Supporting Information

# E3 Ubiquitin ligase ring finger protein 2 alleviates cerebral ischemia/reperfusion injury by stabilizing mesencephalic astrocyte-derived neurotrophic factor through monoubiquitination

**Yujun Shen^1,2^, Jinfeng Wang^1,2^, Junxing Liang^1,2^, Ying Chen^1,2^, Xueyan Wu^1,3^ Zhenhua Ren^1,3^, Jiangning Zhou^3^, Lijie Feng^1,2,3*^, Yuxian Shen^1,2,3*^**

^1^School of Basic Medical Sciences, Anhui Medical University, Hefei 230032, China; ^2^Biopharmaceutical Research Institute, Anhui Medical University, Hefei 230032, China ^3^Anhui Provincial Key Laboratory for Brain Bank Construction and Resource Utilization, Anhui Medical University, Hefei 230032, China

**Materials and Methods**

**Cell culture and treatment.** Human neuroblastoma SH-SY5Y cells and mouse neuroblastoma N2a cells were obtained from the Institute of Cell Biology, Chinese Academy of Sciences (Shanghai, China). These cells were cultured in Dulbecco’s modified Eagle’s medium (DMEM) supplemented with 10% fetal bovine serum, and then placed in a humidified incubator (37°C, 5% CO2). N2a cells were treated with tunicamycin (TM, 2.5 μg/ml) for 16 h or Oxygen-Glucose Deprivation/Reperfusion (OGD/R) to mimic the ischemic attack in vitro. Briefly, cells cultured in medium deprived of serum and glucose were placed in a hypoxic chamber with 95% N2 and 5% CO2. The chamber was then incubated at 37 °C for 1 h, 2 h, 3 h and 4 h, respectively. After OGD, cells were removed from the hypoxic chamber and incubated in glucose-containing medium in an incubator with 95% air and 5% CO2 for 24 h. N2a cells were treated with recombinant human MANF (His-MANF, rhMANF) protein at 0, 5, 10, or 20 μg/mL for 2 h before cell harvesting.

**Plasmids, small interfering RNA (siRNA), and transfection.** The plasmid pCS2-6MT-RNF2 was donated by Professor Aaron Ciechanover from Rapaport Medical College and Research Institute, and pGEX4T-1-RNF2 was donated by Professor Seongman Kang from Korea University. The plasmids PEGFP-C2-RNF2, pCIneo-Vector, pCIneo-MANF-FLAG, pET28a-His-MANF were previously constructed and preserved in our laboratory.^1^ RNF2 and MANF specific siRNAs were purchased from GenePharma (Shanghai, China). Plasmid DNA and siRNA transfection were performed using Lipofectamine 2000 (Invitrogen) according to the manufacturer’s instruction. The sequences of siRNAs were shown in Table S2.

**Adeno-associated viruses (AAV) administration.** Lentiviral expression vector pAAV-CMV-mCherry-P2A-3×FLAG-WPRE for overexpressing RNF2 (AAV-RNF2, 3.36 × 10^13^ vg/ml) were supplied by Obio Technology (Shanghai, China). The empty AAV expression vector coding mCherry (AAV-con) were used as the control. Three weeks before MCAO, AAV were stereotaxically injected into the right lateral ventricle as follows: A/P = -1.6 mm, L/M =+1.5 mm, D/V=−4.5 mm.^2^ Injection was performed at a flow rate of 0.2 μl/min and the optimal injection volume was determined to be 10 μl. After injection, the rats were rested for 3 weeks before further analysis.

**2, 3, 5-Triphenyltetrazolium chloride (TTC) staining.** Rats were decapitated 24 h after MCAO. After perfusion, the brains were immediately removed and sliced into 2.0-mm-thick sections and incubated in 2% TTC solution for 15 min at 37°C, then transferred into a 4% paraformaldehyde solution for fixation. The images were captured with a digital camera and the infarct volumes were quantified with Photoshop software (Adobe, SanJose, CA, USA). The infarct percentage of the four sections was calculated according to our published method.^3^

**Immunohistochemistry.** Immunohistochemistry assay was performed as previously described.^3^ In short, brain tissues were deparaffinized, performed an antigen retrieval, blocked with goat serum, incubated with following antibodies: rabbit anti-RNF2 antibody (1:100, Abcam, ab187509), rabbit anti-caspase 3 (active) (1:400, CST, 9654s) overnight at 4 °C following with biotinylated secondary antibodies incubation. Then, the sections were stained with 3,3’-diaminobenzidine tetrahydrochloride (DAB, ZSGB-BIO, ZLI-9018) and hematoxylin, dehydrated in graded ethanol, cleared in xylene, and then observed under a digital slide scanner and slide viewer software (Pannoramic MIDI and Case Viewer 2.4, 3D Histech, Hungary).

**Immunofluorescent staining.** Immunocytofluorescent staining was performed as described before.^4^ Brain slices were hydrated and rinsed in phosphate buffered saline (PBS). After antigen retrieval, sections were permeabilized in PBS containing 0.5% Triton X-100 and blocked in 5% goat serum. The sections were incubated with primary antibodies overnight at 4°C. For dual or multiple fluorescent staining (Pentuple-Fluorescence immunohistochemical mouse/rabbit kit, immunoway company), the slices were incubated with different Alexa Fluor conjugated secondary antibodies. DAPI was used for nuclei staining. Images were taken using confocal laser scanning microscope (ZEISS, Germany) with constant parameters of acquisition. The involved antibodies were included in Supporting Information Table S4.

**Pentuple-fluorescence immunofluorescent staining.** Pentuple-fluorescence immunofluorescent staining was performed according to the protocol. Put the dewaxing and repair two-in-one reagent into the repair box and heat it to boiling, put the slices in and continue heating for 30 min, then stop heating and cool naturally to room temperature. Take out the slices and wash them in distilled water for 6 times, then add 100 μl peroxidase blocking buffer incubate at room temperature for 15 min, and wash with PBST for 6 min. Then add anti-NeuN antibody, incubate at 37°C for 2 h, and wash with PBST for 6 min. Then add 100 μl of HRP multipolymer anti-rabbit/ mouse secondary antibody, incubate at room temperature for 30 min, and wash with PBST for 6 min. Then add 100 μl of fluorescent dye, incubate at room temperature for 10 min, and wash with PBST for 6 min. Then add antibody stripping solution, heat for 15 min, cool naturally, and wash with PBST for 6 min. Repeat the step of adding the primary antibody to the antibody peel solution. Finally, DAPI solution is added to avoid bubbles. Cover the slides and scan them with 3D histech Pannoramiic MIDI.

**TUNEL assay.** The apoptotic cells were detected in TM-treated cells or MCAO brain slices using the TUNEL kit (Beyotime) following the manufacturer's instructions. Images were captured on a Zeiss microscope imaging system (Zeiss, LSM 800 with Airyscan2, Germany) and analyzed by Image J. The numbers of TUNEL-positive cells were counted in 5 randomly selected fields of high-magnification (400×) in each section.

**Flow cytometry with Annexin V/PI double staining.** Apoptosis was assessed by flow cytometric analysis (BD Biosciences, USA). The cells were collected and fixed in 70% ethanol at −20°C for 24 h, and then were stained with Annexin V and propidium iodide (PI) using Annexin V-FITC/PI Apoptosis Detection kit (Bestbio, China) according to the manufacturer’s instructions. Finally, the apoptotic index was measured by flow cytometry and analyzed with Flowjo software (Informer Technologies, USA).

**Quantitative real-time PCR analysis (****qRT-PCR).** Total RNA was extracted from cells and brain tissues with TRIzol (Invitrogen, 15596-018), and cDNA was subsequently synthesized using the PrimeScript™ RT reagent kit (Takara, China). Quantitative Real-time PCR was carried out using TB Green Premix Ex Taq™ II (Takara, China) on QuantStudio 5 instrument (Applied Biosystems, USA). All primers were purchased from Sangon Biotech (Shanghai, China) and primer sequences are included in Table S3.

**Western Blot.** Brain tissue and cells were lysed in lysis buffer (250 mmol/L Tris-HCl, pH 7.4, 2.5% SDS). Cell lysates were centrifuged at 14000 g at 4 °C for 10 min and the supernatant was harvested and protein concentration was quantified using the BCA Protein Assay (Thermo Fisher Scientific, Shanghai, China). The protein was loaded onto SDS-PAGE gels and then transferred to PVDF membranes. After blocking with 5% skim milk for 1 h, membranes were incubated overnight at 4°C with the primary antibodies. After washing in TBST, membranes were incubated with corresponding secondary antibodies at room temperature for 1 h. Blots were visualized by the High-sig (Tanon, China) and Femto-sig (Tanon, China) ECL Western Blotting Substrate kit and chemiluminescence system (Clinx Science Instruments Co., Ltd, China). The densitometric analysis was performed using Image J software. The involved antibodies were included in Table S4.

**Co‑immunoprecipitation (Co‑IP).** Protein samples were obtained from cells or brain tissue as described in “Western Blot” section. Protein A/G beads (Thermofisher, USA) mixed with the anti-Myc antibody or red anti-FLAG M2 Affinity Gel (Merck) was used for Co-IP analysis, mouse (G3A1) mAb IgG1 isotype as Control (CST, USA). These agarose beads were mixed with cell lysis and then eluted. The indicated proteins were detected using Western blot. Fc fragment specific secondary antibody (Jackson, USA) was used to avoid overlap of the monoubiquitinated MANF band position with the light chain chain.

**GST pull-down assay.** Recombinant human MANF (His-MANF, rhMANF) was purified by Ni-NTA Agarose (Invitrogen, USA) and dissolved in PBS buffer. Then, His-MANF protein was incubated with appropriate number of Pierce Glutathione Agarose (Thermo fisher, USA) bearing immobilized GST-RNF2 fusion protein or GST protein at 4 ℃ for 2 h. The pulled down proteins were detected by Coomassie brilliant blue and western blot with anti-MANF antibody (Abcam, USA).

***In vitro* ubiquitination assay.** Using the rabbit reticulocyte lysate (RRL) (Promega, USA) as a source of E1 and E2, the ubiquitination assay of GST-RNF2 was performed as described previously.^5^ Briefly, the ubiquitination buffer containing 40 mM Tris-HCl, pH 7.5, 5 mM MgCl_2_, 2 mM ATP, 2 mM dithiothreitol (DTT), 300 ng/μl ubiquitin (Merck, USA), 25 μM MG132 , 25 μl RRL and 600 ng GST or GST-RNF2 was incubated in 40 μl ubiquitination buffer at 30 °C for 2.5 h. Anti-MANF immunoprecipitates obtained from N2a cell lysates were used as substrates for *in vitro* ubiquitination assay as described previously.^6^ The precipitates were washed five times with lysis buffer, once with ubiquitination buffer and subjected to *in vitro* ubiquitination. Finally, all samples were subjected to western blot with anti-MANF, anti-Ub, anti-RNF2 and anti-GST (Santa Cruz, USA) antibodies.

**Statistical Analysis.** Statistical analysis was performed with SPSS 26.0 software (IBM Corporation) and GraphPad Prism 8.0 software (GraphPad, San Diego, USA). All the date were expressed as mean ± standard deviation (SD). Two-tailed Student’s t-test was used to analyze the difference between two groups and one- way ANOVA followed by Tukey’s post hoc test was used for multiple comparisons. P < 0.05 indicates the significant difference.

**REFERENCES**

1. Apostolou A, Shen Y, Liang Y, et al. Armet, a UPR-upregulated protein, inhibits cell proliferation and ER stress-induced cell death. Exp Cell Res. 2008; 314(13): 2454-2467.
2. Renko JM, Mahato AK, Visnapuu T, et al. Neuroprotective Potential of a Small Molecule RET Agonist in Cultured Dopamine Neurons and Hemiparkinsonian Rats. J Parkinsons Dis. 2021; 11(3): 1023-1046.
3. Yang W, Shen Y, Chen Y, et al. Mesencephalic astrocyte-derived neurotrophic factor prevents neuron loss via inhibiting ischemia-induced apoptosis. J Neurol Sci. 2014; 344(1-2): 129-138.
4. Xu S, Di Z, He Y, et al. Mesencephalic astrocyte-derived neurotrophic factor (MANF) protects against Abeta toxicity via attenuating Abeta-induced endoplasmic reticulum stress. J Neuroinflammation. 2019; 16(1): 35.
5. Liu C, Zhang D, Shen Y, et al. DPF2 regulates OCT4 protein level and nuclear distribution. Biochim Biophys Acta. 2015; 1853(12): 3279-3293.
6. Paolini R, Molfetta R, Piccoli M, et al. Ubiquitination and degradation of Syk and ZAP-70 protein tyrosine kinases in human NK cells upon CD16 engagement. Proc Natl Acad Sci U S A. 2001; 98(17): 9611-9616.

**Figure S1 RNF2 mRNA levels is upregulated in the focal cerebral ischemic rat brain tissues.** MCAO was performed for 2 h occlusion followed reperfusion for 24 h. (A) RNF2 mRNA levels in I/R brain tissues were detected by Reverse Transcription PCR. (B) Quantitation of data as in A (n = 6; ***P＜0.001 vs sham; t-test).

**Figure S2 Intracerebral ventricular injection of adeno-associated virus to overexpressing RNF2 in the ischemic cortex of rats.** (A) Three weeks before MCAO, AAV were stereotaxically injected into the right lateral ventricle as follows: A/P = -1.6 mm, L/M = +1.5 mm, D/V = −4.5 mm. AAV successfully infected rat brain tissue and successfully expressed mCherry protein in brain tissue. Scale bar = 1500 μm. (B) Brain samples were collected from the sham and ischemic rat brain tissue with AAV-RNF2 injection three weeks before I/R. The red fluorescent protein mCherry was expressed in the rat cortex, indicating that AAV-RNF2 can express RNF2 protein in the ischemic cortex. Scale bar = 50 μm. (C) Immunofluorescence staining detected that AAV virus can infect neurons. NeuN-positive cells were detected by immunofluorescence staining with anti-NeuN antibody. mCherry is the virus's own fluorescence. Scale bar = 20 μm.

**Figure S3** **RNF2 expression was acutely upregulated in AAV-RNF2-infected rat brain.** AAV were stereotaxically injected into the lateral ventricle three weeks before I/R, AAV are successfully transfected into nerve cells and effectively alter the expression of RNF2. (A) RNF2 expression were detected by immunohistochemistry. Upper panel scale bar = 50 μm, lower panel scale bar = 20 μm. (B) Quantitation of data as in A. (n = 6; **P < 0.01, ***P < 0.001 vs AAV-Con; t-test)

**Figure S4 AAV-mediated RNF2 overexpression inhibits I/R-induced neuron apoptosis.** AAV were stereotaxically injected into the lateral ventricle three weeks before I/R, AAV are successfully transfected into nerve cells and effectively alter the expression of RNF2. (A) Stably expressing RNF2 with mCherry tag inhibits caspase-3 activation detected by immumohistochemical staining and immunofluorescent staining using antibody against c-Casp-3 (green) in ischemic cerebral cortex. Scale bar = 50 μm. (B, C) Quantitation of data as in A. (n = 6; **P < 0.01, ***P < 0.001; one-way ANOVA followed by Tukey’s test).

**Figure S5 RNF2 protects against TM-induced nerve cells apoptosis.** N2a cells were transiently transfected with the plasmids and siRNA as indicated. After 36 h of transfection, the cells were treated with TM (2.5 μg/ml) for 16 h. (A) Flow cytometry showing apoptotic N2a cells. (B) Quantitation of data as in A. (n = 3; *P < 0.05, ***P < 0.001 vs Myc-Vector or NC; t-test).

**Figure S6** **RNF2 inhibits neuronal cells apoptosis dependently on MANF.** RNF2-Myc plasmid was transfected into MANF knockdown N2a cells, or corresponding controls and treated with TM for 16 h at 36 h posttransfection. N2a cells were treated with rhMANF for 2 h before harvesting. (A) Apoptosis as detected by TUNEL assays. Scale bar = 20 μm. Magnified photo scale bar = 10 μm. (B) Quantitation of data as in A (n = 3; **P＜0.01, ***P＜0.001; one-way ANOVA followed by Tukey’s test).

**Figure S7 AAV-mediated RNF2 overexpression improves the Zea Longa scores.** (A) The time–effect relationship of AAV-mediated RNF2 overexpression on Zea Longa score. AAV-Con or AAV-RNF2 were stereotaxically injected into the lateral ventricle three weeks before I/R. Zea Longa score was evaluated at 24 h and on the days 2, 4, 6, 8, 10, 12 and 14 after I/R. Data are presented as the means ± SEM. (n = 8; *P＜0.05 vs AAV-Con; t-test). (B) Zea Longa score was evaluated at 24 h after I/R. Data are presented as the means ± SEM. (n = 10; *P＜0.05, ****P < 0.0001; one-way ANOVA followed by Tukey’s test). (C) Zea Longa score was evaluated at 4 days after I/R. Data are presented as the means ± SEM. (n = 5; *P＜0.05, ***P < 0.001; one-way ANOVA followed by Tukey’s test). (D) Zea Longa score was evaluated at 10 days after I/R. Data are presented as the means ± SEM. (n = 4; *P＜0.05, ***P < 0.001; one-way ANOVA followed by Tukey’s test). (E) Zea Longa score was evaluated at 14 days after I/R. Data are presented as the means ± SEM. (n = 4; *P＜0.05, **P < 0.01; one-way ANOVA followed by Tukey’s test).

**Figure S8 OGD/R treatment increases the expression of RNF2 and MANF in SH-SY5Y cells.** (A) SH-SY5Y cells were treated with OGD/R for 3 h and the cells were collected after 24 h of oxygen-glucose reoxygenation. Western blot was used to detect the level of RNF2 and MANF. (B) Quantitation of data as in A (n = 3; ***P＜0.001 vs Mock; t-test). (C) Repeat 3 dishes of cells as in A. (D) Quantitation of data as in C (n = 3; ***P＜0.001 vs Mock; t-test).

**Figure S9 RNF2 regulates MANF protein level but not transcription level in SH-SY5Y cells with OGD/R treatment.** SH-SY5Y cells were transfected with RNF2-siRNA or RNF2-Myc, and treated with OGD/R at 36 h after transfection. Mock means without OGD/R treatment. The protein levels of RNF2 and MANF were detected with anti-RNF2 and anti-MANF antibodies, respectively. Tubulin was used as a loading control. (B) Quantitation of data as in A. (n = 4; ***P＜0.001 vs NC or Myc-Vector; t-test). (C) RNF2 upregulates MANF in a dose-dependent manner. SH-SY5Y cells were transiently transfected with RNF2-Myc plasmid (0 μg, 0.25 μg, 0.5 μg, 1.0 μg). After 36 h of transfection, the cells were treated with OGD/R. The level of MANF and RNF2 were detected by western blot assay. Tubulin was used as a loading control. (D) Variation curve of MANF level in the C. (E) RNF2 had no effect on MANF transcription level. SH-SY5Y cells were transfected with RNF2-siRNA or RNF2-Myc for 36 h. The expression of RNF2 and MANF in SH-SY5Y cells were detected by using relative quantitative RT-PCR assay. GAPDH was used as loading control. (F) Quantitation of data as in E (n = 3; **P＜0.01, ***P＜0.001 vs NC or Myc-Vector; t-test).

**Figure S10 RNF2 interacts with MANF in SH-SY5Y cells.** SH-SY5Y cells were transfected with RNF2-Myc or RNF2-siRNA, and treated with OGD/R at 36 h after transfection. (A, C) RNF2 (red) and MANF (green) was detected by immunofluoscent staining in SH-SY5Y cells. DAPI was used to counterstain the nuclei. The scale bar = 10 μm. (B, D) Quantitation of data as in A and C. Percentage of RNF2 and MANF colocalization cells in A and B. (n = 6; **P＜0.01, ***P＜0.001, ***P＜0.0001 vs Myc-Vector or NC; t-test).

**Figure S11 RNF2 inhibits neuronal cells apoptosis dependently on MANF.** SH-SY5Y cells were transfected with RNF2-Myc or RNF2-siRNA, and treated with OGD/R at 36 h after transfection. (A) The proteins were detected with anti-bcl-2, anti-bax and anti-cleaved caspases-3 antibody, respectively. Tubulin was used as a loading control. (B) Quantitation of data as in A (n = 3; **P＜0.01, ***P＜0.001 vs NC or Myc-Vector; t-test). (C) Bax/Bcl-2 ratio was evaluated as in A (n = 3; **P＜0.01, ***P＜0.001 vs NC or Myc-Vector; t-test). (D) RNF2 overexpression protects against OGD/R-induced nerve cells apoptosis. SH-SY5Y cells were transiently transfected with the plasmids and siRNA as indicated. After 36 h of transfection, the cells were treated with OGD/R. (D) Flow cytometry showing apoptotic SH-SY5Y cells. (E) Quantitation of data as in D. (n = 3; ****P < 0.0001 vs Myc-Vector or NC; t-test).

**Figure S12 RNF2 inhibits neuronal cells apoptosis dependently on MANF.** RNF2-Myc plasmid was transfected into MANF knockdown SH-SY5Y cells, or corresponding controls and treated with OGD/R at 36 h posttransfection. SH-SY5Y cells were treated with rhMANF for 2 h before harvesting. (A) Flow cytometry showing apoptotic SH-SY5Y cells cells. (B) Quantitation of data as in A (n = 3; *P＜0.05, ***P＜0.001, ****P < 0.0001; one-way ANOVA followed by Tukey’s test). (C) Apoptosis as detected by TUNEL assays. Scale bar = 20 μm. Magnified photo scale bar = 10 μm. (D) Quantitation of data as in C (n = 3; **P＜0.01, ***P＜0.001; one-way ANOVA followed by Tukey’s test).

**Table S1 Biological Samples utilized in the study.**

| **Sample No.** | **Age/sex (y)** | **Time of death** | | **Postmortem interval (min)** | **Medical history** | **Cause of death** | |
| --- | --- | --- | --- | --- | --- | --- | --- |
| Control #1 | 82/M | 2021/2/17 3:00 | | 480 | hypertension | unknown | |
| Control #2 | 69/M | 2023/11/14 21:31 | | 199 | Prostate tumors | respiratory failure | |
| Control #3 | 91/M | 2023/12/8 17:00 | | 390 | heart disease | unknown | |
| Stroke #1 | 78/M | 2021/1/18 17:00 | | 120 | stroke, PD | severe pneumonia | |
| Stroke #2 | 75/M | 2023/11/2 20:27 | | 183 | hypertension, Cerebral thrombosis, stroke, cerebral hemorrhage, severe pneumonia | unknown | |
| Stroke #3 | 92/M | 2023/11/8 14:00 | | 295 | hypertension, diabetes, stroke | unknown | |
| **Table S2 Oligonucleotides sequences of siRNAs utilized in the study.** | | | | | | | |
| *si-RNF2#1* | | | | GCAGACAAAUGGAACUCAACCAUUA | | | |
| *si-RNF2#2* | | | | UUAAUUCACUGUGUAGACUUCUAGG | | | |
| *si-RNF2#3* | | | | CCUAGAAGUCUACACAGUGAAUUAA | | | |
| *si-MANF#1* | | | | GGACCUCAAAGACAGAGAUTT | | | |
| *si-MANF#2* | | | | GCAGAUCGACCUGAGCACATT | | | |

**Table S3 Primer sequences used for PCR.**

| **Primer** | **PCR** | **Forward** | **Reverse** |
| --- | --- | --- | --- |
| Rat RNF2 | qRT | TGGATGGTGCCAGTGAGATT | AACAGTGGCATTGCCTGAAG |
| Rat GAPDH | qRT | GACATGCCGCCTGGAGAAAC | AGCCCAGGATGCCCTTTAGT |
| Rat RNF2 | Relative qPCR | CTCAGGCTGTGCAGACAAAT | GATCCTAGCTAATACTCTCT |
| Rat GAPDH | Relative qPCR | CCACTCCTCCACCTTTG | CACCACCCTGTTGCTGT |
| RAT MANF | Relative qPCR | TCACATTYTCACCAGCCACT | ATCTGGCTGTCYTTCTTCTTMA |

**Table S4 Key resources used in the study.**

| **REAGENTorRESOURCE** | **SOURCE** | **IDENTIFIER** |
| --- | --- | --- |
| **Antibodies** |  |  |
| Rabbit anti-RNF2 | Origene | Cat# TA332768; Lot# P00009 |
| Rabbit anti-RNF2 | Abcam | Cat# AB101273; Lot# GR48080-1, GR44360-1, GR64557-1 |
| Rabbit anti-RNF2 | CST | Cat# 5694S; Lot# 1 |
| Rabbit anti-RNF2 | Abcam | Cat# AB187509; Lot# GR313657-4, 1026309-2 |
| Mouse anti-RNF2 | Affinity | Cat# BF8076; Lot# 27Z0747 |
| Rabbit anti-NeuN | Abcam | Cat# ab177487; Lot# GR249899-78 |
| Rabbit anti-GFAP | Proteintech | Cat# 16825-1-AP; Lot# 00132923 |
| Rabbit anti-CD68 | Affinity | Cat# DF7518; Lot# 61I9296 |
| Rabbit anti-CNP | Proteintech | Cat#13427-1-AP; Lot# 020 |
| Rabbit anti-caspase 3 (active) (IHC) | CST | Cat# 9664S; Lot# 20 |
| Mouse anti-FLAG M2 | Merck | Cat# F1804; Lot# 1003269627 |
| Mouse anti-MYC | CST | Cat# 2276S; Lot# 19 |
| Rabbit anti-GST | Santa Cruz | Cat# SC-292368; Lot# G2611 |
| Rabbit anti-GFP | Abcam | Cat# ab290; Lot# GR194813-1 |
| Mouse anti-GFP | Abm | Cat# G096; Lot# 0511 |
| Rabbit anti-GFP | Proteintech | Cat# 50430-2-AP; Lot# 0551 |
| Rabbit anti-mCherry | CST | Cat# 43590S; Lot# 3 |
| Mouse anti-mCherry | Abcam | Cat# AB125096; Lot# 1014690-1 |
| Rabbit anti-MANF | Abcam | Cat# AB67271; Lot# 1020345-10 |
| Rabbit anti-MANF | Abcam | Cat# AB253243; Lot# GR3272138-4 |
| Mouse anti-MANF | Santa Cruz | Cat# SC-515907; Lot# E3117 |
| Rabbit anti-BIP | Abcam | Cat# AB53068; Lot# GR6512-8 |
| Rabbit anti-BIP | Proteintech | Cat# 11587-1-AP; Lot# 010 |
| Rabbit anti-H3 | Abcam | Cat# AB70550; Lot# GR26365-1, GR36063-1 |
| Mouse anti-H3 | Affinity | Cat# BF9211; Lot# 21e7638 |
| Rabbit anti-CHOP | Santa Cruz | Cat# SC-793; Lot# C2612 |
| Mouse anti-ubiquitin | Santa Cruz | Cat# SC-8017; Lot# I2413 |
| Rabbit anti-ubiquitin | Abcam | Cat# AB134953; Lot# GR3367020-12 |
| Mouse anti-α-tubulin | Merck | Cat# T6199; Lot# 064M4811V |
| Goat anti-Rabbit | Elabscience | Cat# E-AB-1003; Lot# AB18519 |
| Goat anti-Mouse | Elabscience | Cat# E-AB-1001; Lot# AB18518 |
| Mouse (G3A1) mAb IgG1 isotype | CST | Cat# 5415S; Lot# 10 |
| Goat Anti-Rabbit IgG, Fc Fragment Specific | Jackson | Cat# 111-035-008; Lot# 138950 |
| Goat anti-rabbit (Alexa Fluor 488 Conjugate) | CST | Cat# 4412; Lot# 25 |
| Goat anti-mouse (Alexa Fluor 488 Conjugate) | CST | Cat# 4408; Lot# 22 |
| Goat anti-rabbit (Alexa Fluor® 555 Conjugate) | CST | Cat# 4413; Lot# 23 |
| Goat anti-mouse (Alexa Fluor® 555 Conjugate) | CST | Cat# 4409; Lot# 21 |
| **Virus strains** |  |  |
| pAAV-CMV-mCherry-P2A-3×FLAG-WPRE | OBiO | Cat# A0V025-AAV2/9; Lot# TS0612 |
| pAAV-CMV-Rnf2-3×FLAG-P2A-mCherry-WPRE | OBiO | Cat# H25175-AAV2/9; Lot# TS0703 |
| **Chemicals, peptides and recombinant proteins** |  |  |
| Dulbecco’s modified Eagle’s medium | Gibco | Cat# 11965092 |
| Fetal bovine serum | Gibco | Cat# 10099141 |
| 2,3,5-Triphenyltetrazolium chloride | Sigma | Cat# T8877 |
| Tunicamycin | Millipore | Cat# 654380-10MG |
| 4′,6-diamidino-2-phenylindole | Sigma | Cat# D9564 |
| Pentuple-Fluorescence immunohistochemical mouse/rabbit kit | Immunoway | Cat# RS0038 |
| Annexin V-APC/PI kit | Bestbio | Cat# BB-41033-100T |
| TRIzol | Invitrogen | Cat# 15596-018 |
| PrimeScript™ RT Master Mix | TAKARA Biotechnology LTD | Cat# RR037A |
| Ex Taq polymerase | TAKARA Biotechnology LTD | Cat# RR902A |
| TB Green Premix Ex Taq™ II | TAKARA Biotechnology LTD | Cat# RR820A |
| Chemistar ^TM^ High-sig ECL Western Blotting Substrate | Tanon | Cat# 180-5001 |
| Tanon ^TM^ Femto-sig ECL Western Blotting Substrate | Tanon | Cat# 180-506 |
| Protein A/G beads | Thermofisher | Cat# 20421 |
| Red mouse anti-FLAG M2 Affinity Gel | Merck | Cat# F2426 |
| Ni-NTA Agarose | Invitrogen | Cat# R90101 |
| Pierce Glutathione Agarose | Thermo Scientific | Cat# 16100 |
| 3,3’-diaminobenzidine tetrahydrochloride | ZSGB-BIO | Cat# ZLI-9018 |
| TUNEL Cell Apoptosis Detection Kit (Green Fluorescence) | Beyotime | Cat# C1086 |
| TUNEL Cell Apoptosis Detection Kit (Red Fluorescence) | Beyotime | Cat# C1089 |
| Ubiquitin | Merck | Cat# U5507 |
| Annexin V-FITC/PI Apoptosis Detection kit | Bestbio, China | Cat# BB-41033-100T |
| Pierce BCA Protein Assay Kit | Thermo Fisher Scientific | Cat# 23225 |
| Rabbit reticulocyte lysate | Promega | Cat# L4151 |
| **Software and algorithms** |  |  |
| Chemiluminescence system | Clinx Science Instruments Co., Ltd, China | Model Q4600; https://www.clinx.cn/ |
| Image J | NIH | https://imagej.net/software/fiji |
| Adobe Photoshop | Adobe | 2024 version; https://www.adobe.com/products/photoshop.html |
| Digital slide scanner and slide viewer software | 3D Histech, Hungary | Model pannoramic MIDI and Case Viewer 2.4 |
| Confocal laser scanning microscope | ZEISS, Germany | Model ZEISS 800 |
| Flow cytometry | BD Biosciences, USA | Model FACS Celesta |
| PCR instrument | Bio-rad | Model T100 |
| qRT-PCR instrument | Applied Biosystems, USA | Model QuantStudio 5 |
| SPSS 17.0 | San Diego, USA | https://www.ibm.com/spss |
| GraphPad Prism 8.3.0 | San Diego, USA | https://www.graphpad.com |
