# Supplementary figures and images for: E3 Ubiquitin Ligase Ring Finger Protein 2 Alleviates Cerebral Ischemia–Reperfusion Injury by Stabilizing Mesencephalic Astrocyte‐Derived Neurotrophic Factor Through Monoubiquitination
Source: CNS Neurosci Ther. 2024 Nov 30;30(11):e70136. doi: 10.1111/cns.70136 (PMC11607471; doi:10.1111/cns.70136)

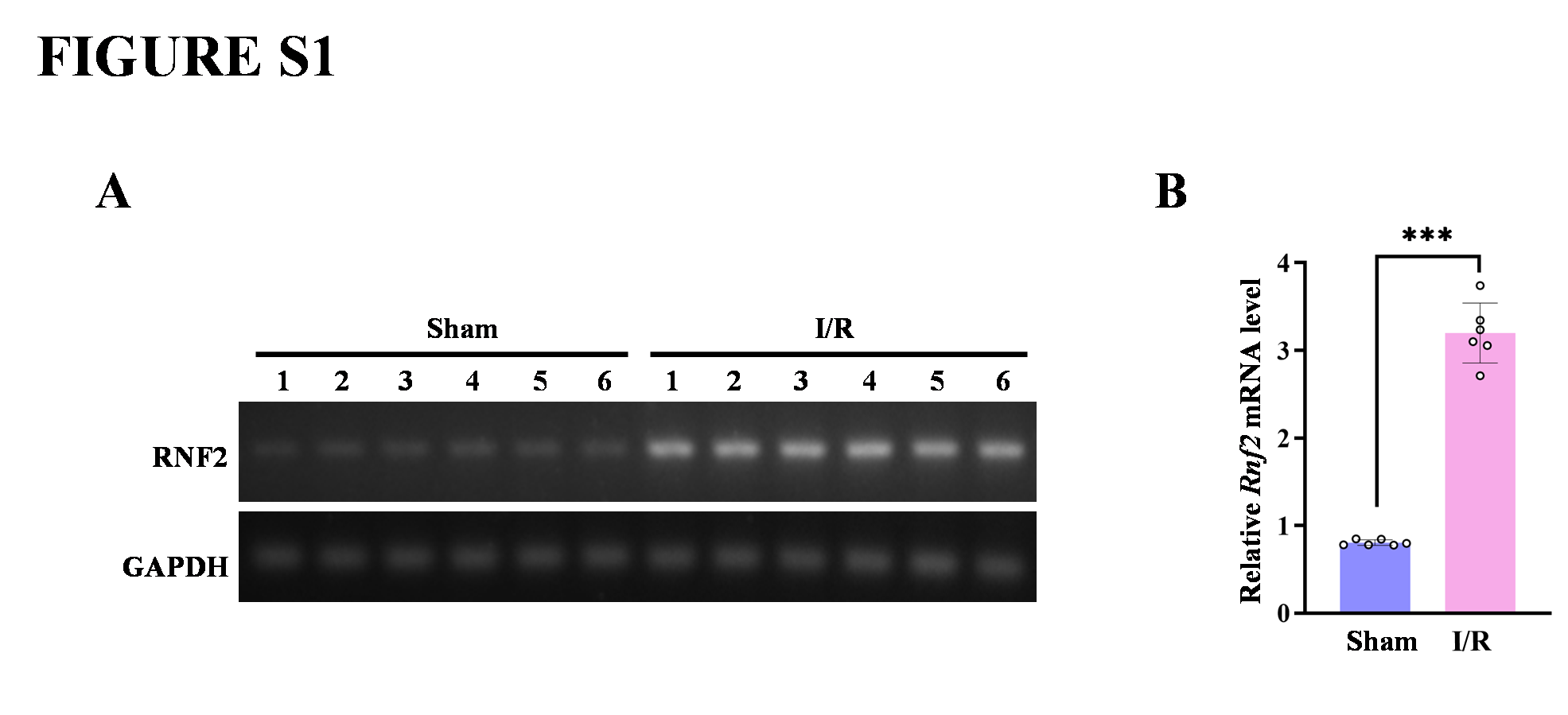

Supplement: Supplementary file 2 — Figure S1. RNF2 mRNA levels are upregulated in the focal cerebral ischemic rat brain tissues. MCAO was performed for 2 h occlusion followed by reperfusion for 24 h. (A) RNF2 mRNA levels in I/R brain tissues were detected by reverse transcription PCR. (B) Quantitation of data as in A (n = 6; ***p < 0.001 vs. sham; t‐test). [file CNS-30-e70136-s006.tif]

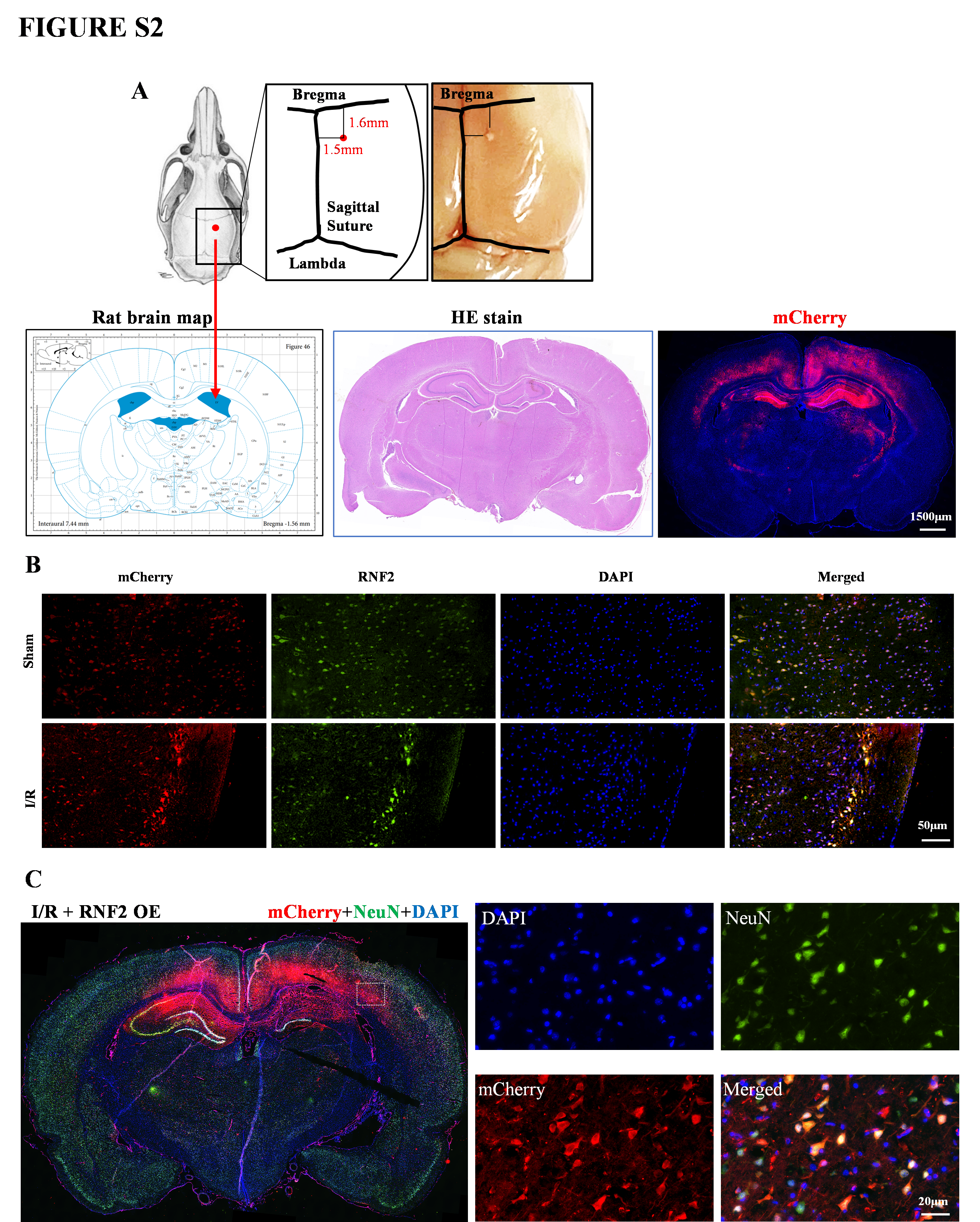

Supplement: Supplementary file 3 — Figure S2. Intracerebral ventricular injection of adeno‐associated virus to overexpressing RNF2 in the ischemic cortex of rats. (A) Three weeks before MCAO, AAV were stereotaxically injected into the right lateral ventricle as follows: A/P = −1.6 mm, L/M = +1.5 mm, D/V = −4.5 mm. AAV successfully infected rat brain tissue and successfully expressed mCherry protein in brain tissue. Scale bar = 1500 μm. (B) Brain samples were collected from the sham and ischemic rat brain tissue with AAV‐RNF2 injection 3 weeks before I/R. The red fluorescent protein mCherry was expressed in the rat cortex, indicating that AAV‐RNF2 can express RNF2 protein in the ischemic cortex. Scale bar = 50 μm. (C) Immunofluorescence staining detected that AAV can infect neurons. NeuN‐positive cells were detected by immunofluorescence staining with the anti‐NeuN antibody. mCherry is the virus’s own fluorescence. Scale bar = 20 μm. [file CNS-30-e70136-s002.tif]

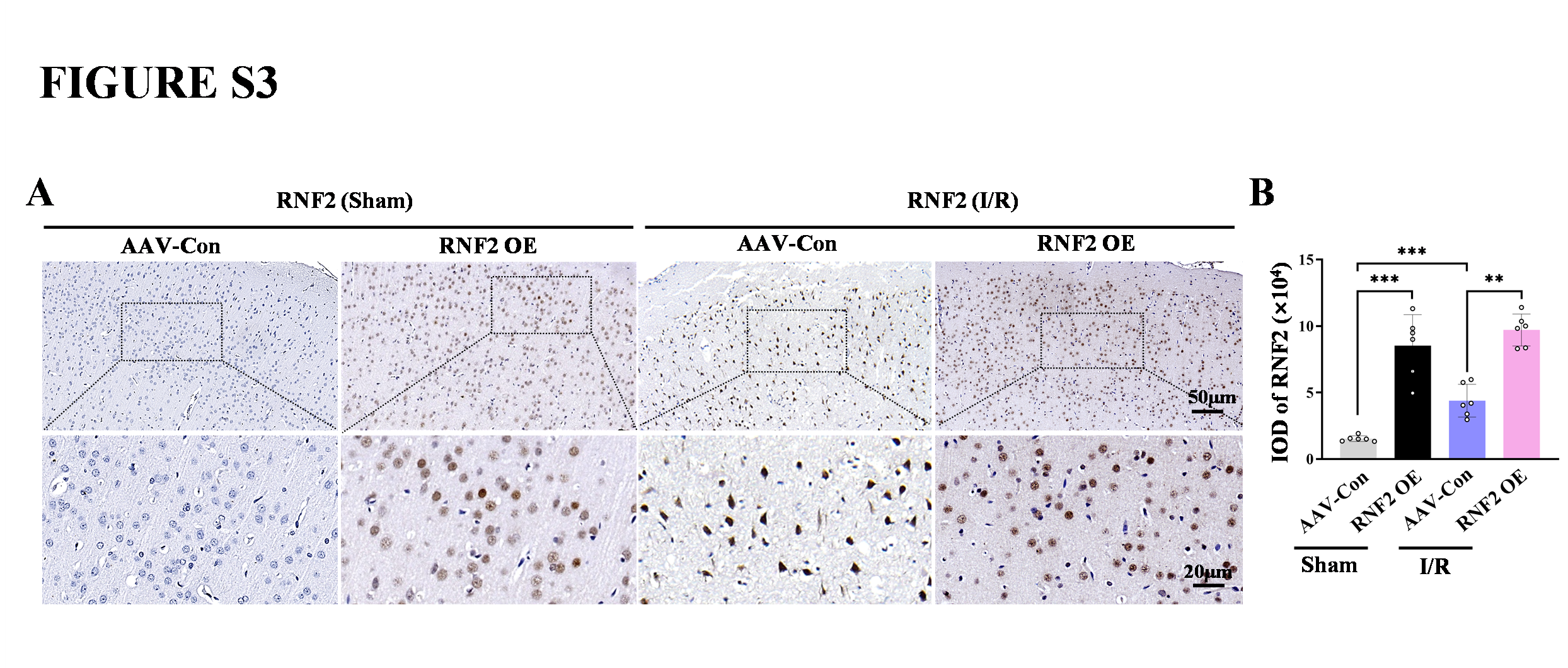

Supplement: Supplementary file 4 — Figure S3. RNF2 expression was acutely upregulated in the AAV‐RNF2‐infected rat brain. AAV were stereotaxically injected into the lateral ventricle 3 weeks before I/R; AAV are successfully transfected into nerve cells and effectively alter the expression of RNF2. (A) RNF2 expression was detected by immunohistochemistry. Upper panel scale bar = 50 μm, lower panel scale bar = 20 μm. (B) Quantitation of data as in A. (n = 6; **p < 0.01, ***p < 0.001 vs. AAV‐Con; t‐test). [file CNS-30-e70136-s007.tif]

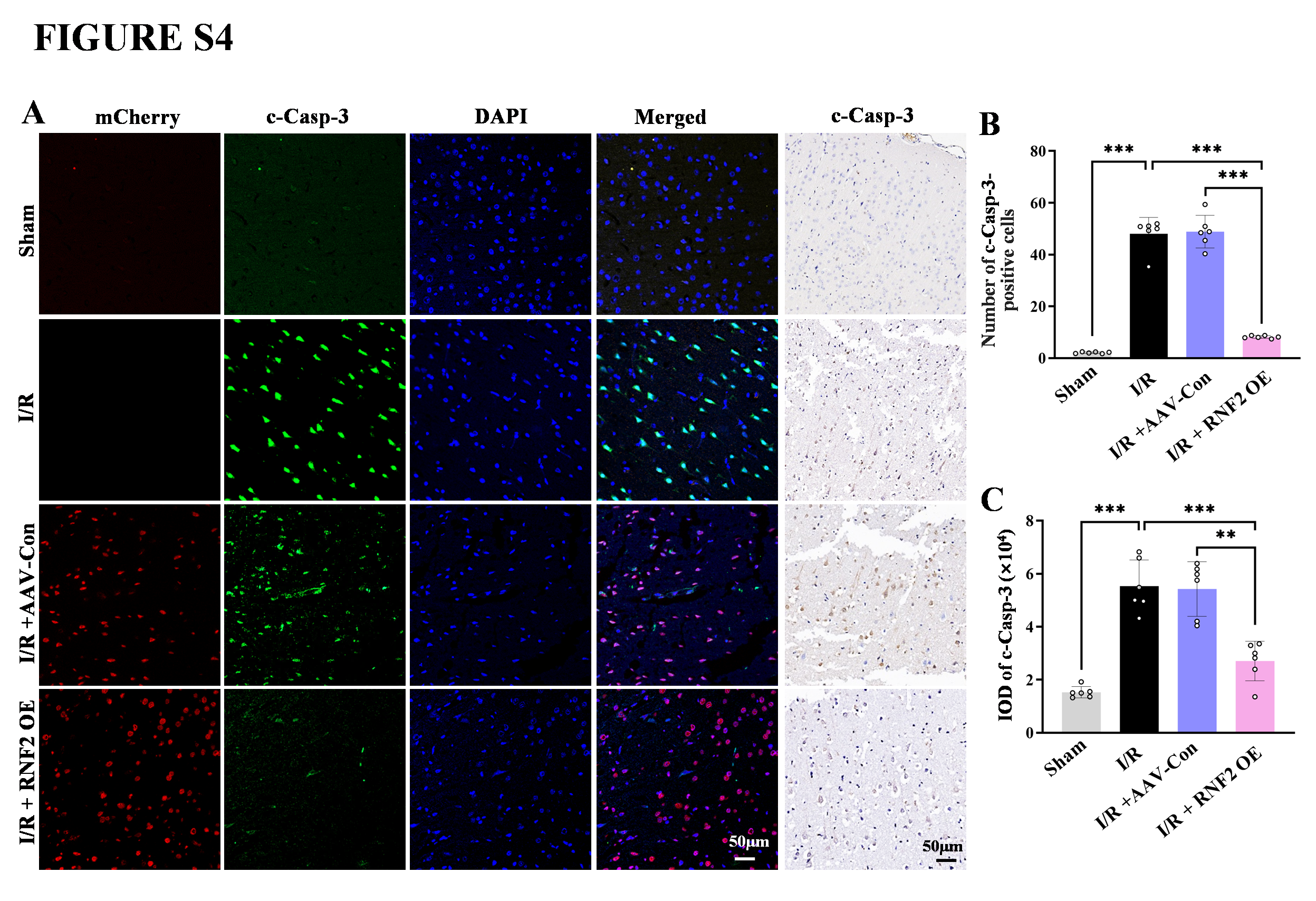

Supplement: Supplementary file 5 — Figure S4. AAV‐mediated RNF2 overexpression inhibits I/R‐induced neuron apoptosis. AAV were stereotaxically injected into the lateral ventricle 3 weeks before I/R; AAV are successfully transfected into nerve cells and effectively alter the expression of RNF2. (A) Stably expressing RNF2 with the mCherry tag inhibits caspase‐3 activation detected by immumohistochemical staining and immunofluorescent staining using antibody against c‐Casp‐3 (green) in the ischemic cerebral cortex. Scale bar = 50 μm. (B, C) Quantitation of data as in A. (n = 6; **p < 0.01, ***p < 0.001; one‐way ANOVA followed by Tukey’s test). [file CNS-30-e70136-s001.tif]

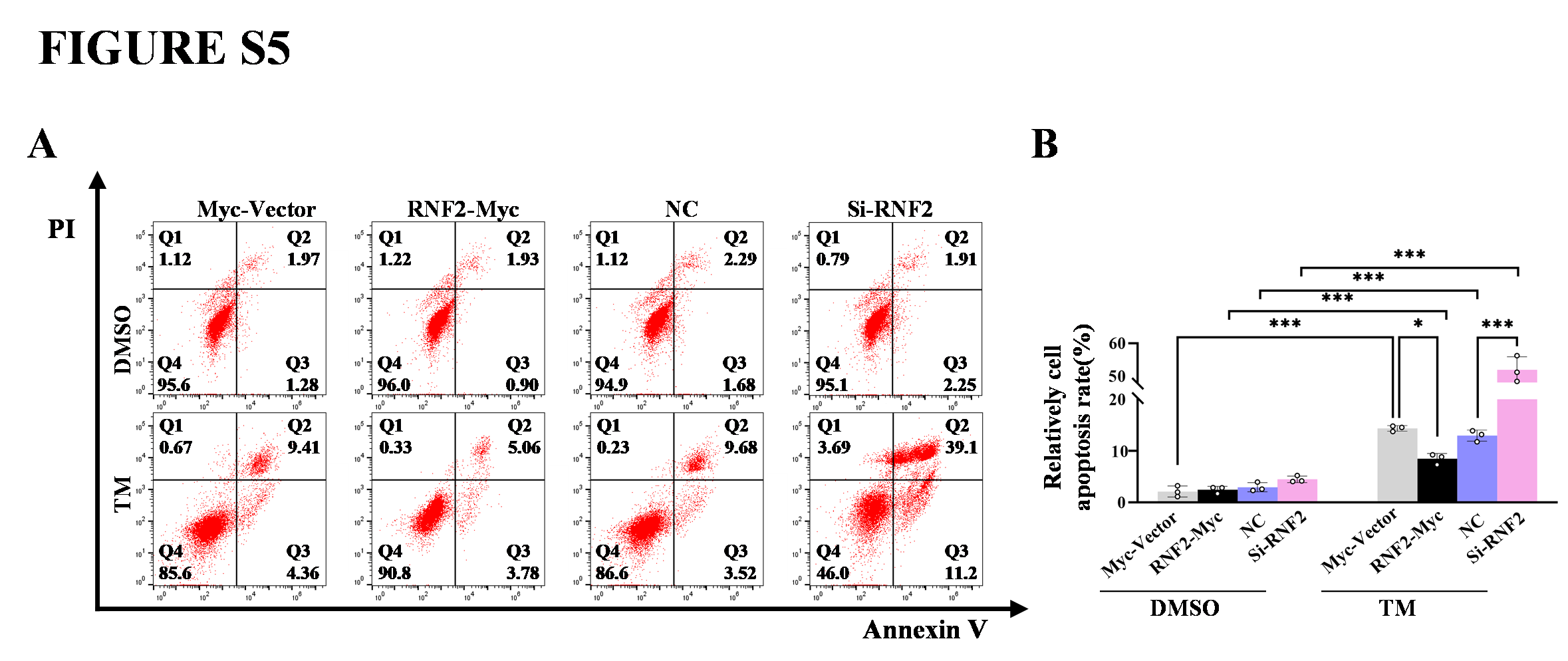

Supplement: Supplementary file 6 — Figure S5. RNF2 protects against TM‐induced nerve cells apoptosis. N2a cells were transiently transfected with the plasmids and siRNA as indicated. After 36 h of transfection, the cells were treated with TM (2.5 μg/mL) for 16 h. (A) Flow cytometry showing apoptotic N2a cells. (B) Quantitation of data as in A. (n = 3; *p < 0.05, ***p < 0.001 vs. Myc‐Vector or NC; t‐test). [file CNS-30-e70136-s004.tif]

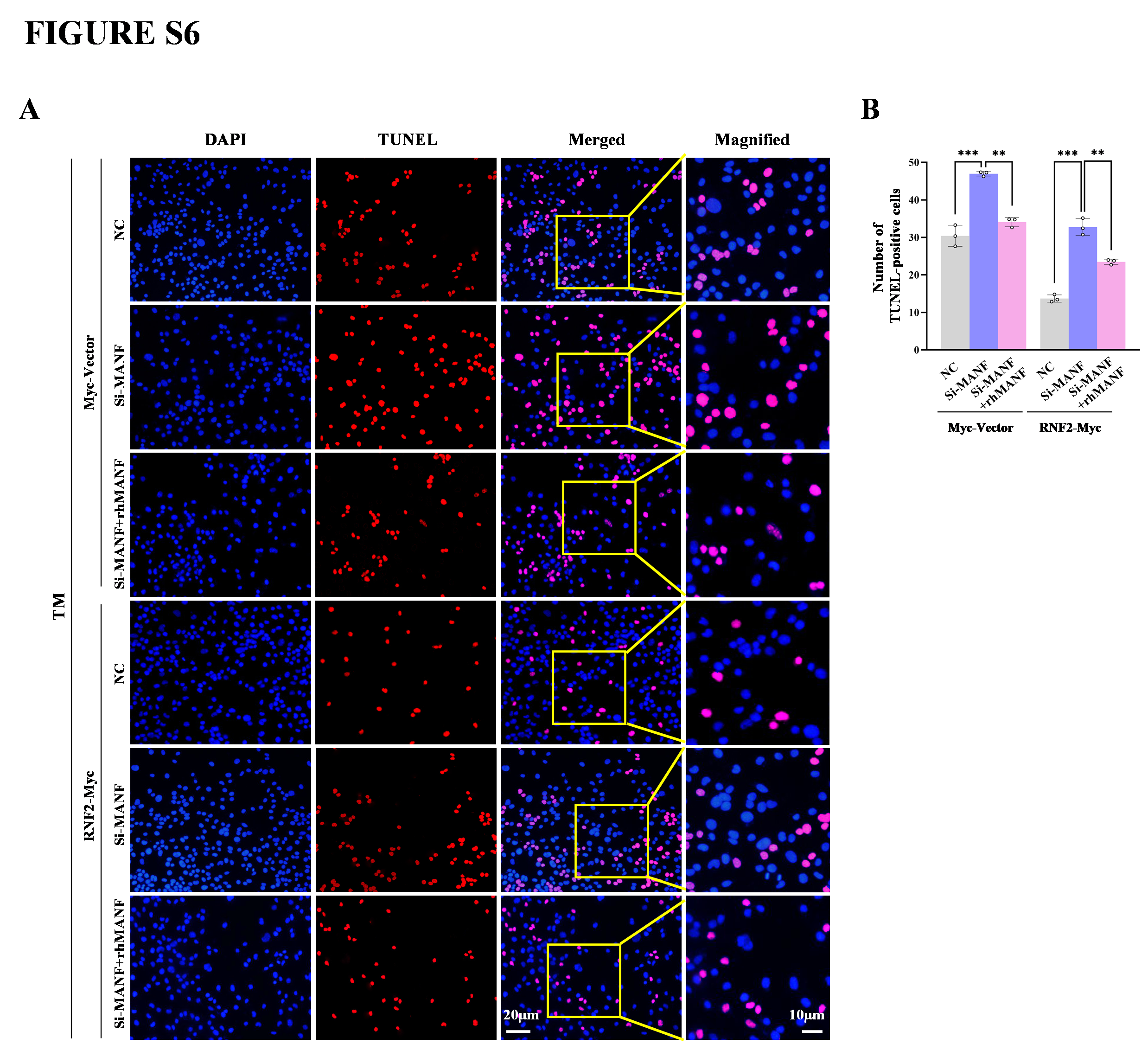

Supplement: Supplementary file 7 — Figure S6. RNF2 inhibits neuronal cells apoptosis dependently on MANF. RNF2‐Myc plasmid was transfected into MANF knockdown N2a cells, or corresponding controls and treated with TM for 16 h at 36 h posttransfection. N2a cells were treated with rhMANF for 2 h before harvesting. (A) Apoptosis as detected by TUNEL assays. Scale bar = 20 μm. Magnified photo scale bar = 10 μm. (B) Quantitation of data as in A (n = 3; **p < 0.01, ***p < 0.001; one‐way ANOVA followed by Tukey’s test). [file CNS-30-e70136-s013.tif]

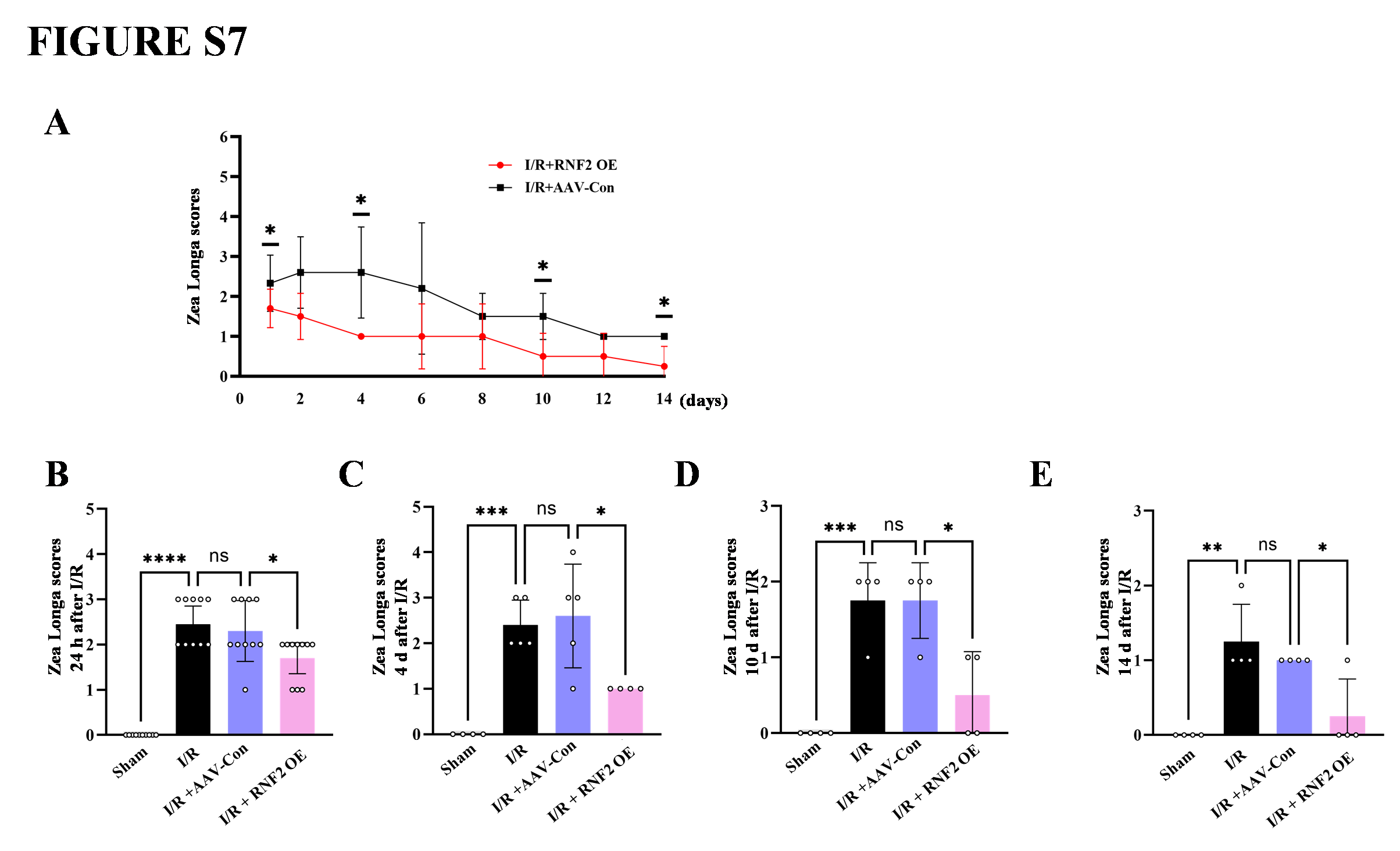

Supplement: Supplementary file 8 — Figure S7. AAV‐mediated RNF2 overexpression improves the Zea Longa scores. (A) The time–effect relationship of AAV‐mediated RNF2 overexpression on the Zea Longa score. AAV‐Con or AAV‐RNF2 were stereotaxically injected into the lateral ventricle 3 weeks before I/R. The Zea Longa score was evaluated at 24 h and on the days 2, 4, 6, 8, 10, 12, and 14 after I/R. Data are presented as the means ± SEM. (n = 8; *p < 0.05 vs. AAV‐Con; t‐test). (B) Zea Longa score was evaluated at 24 h after I/R. Data are presented as the means ± SEM. (n = 10; *p < 0.05, ****p < 0.0001; one‐way ANOVA followed by Tukey’s test). (C) Zea Longa score was evaluated at 4 days after I/R. Data are presented as the means ± SEM. (n = 5; *p < 0.05, ***p < 0.001; one‐way ANOVA followed by Tukey’s test). (D) Zea Longa score was evaluated at 10 days after I/R. Data are presented as the means ± SEM. (n = 4; *p < 0.05, ***p < 0.001; one‐way ANOVA followed by Tukey’s test). (E) Zea Longa score was evaluated at 14 days after I/R. Data are presented as the means ± SEM. (n = 4; *p < 0.05, **p < 0.01; one‐way ANOVA followed by Tukey’s test). [file CNS-30-e70136-s011.tif]

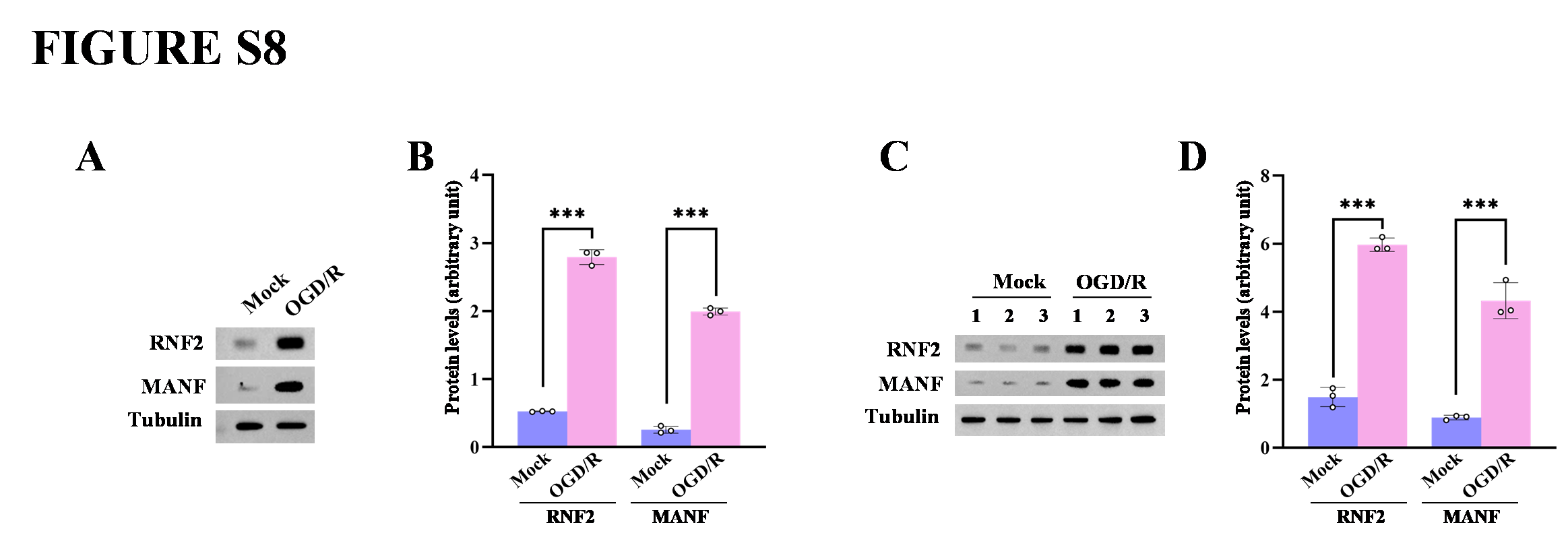

Supplement: Supplementary file 9 — Figure S8. OGD/R treatment increases the expression of RNF2 and MANF in SH‐SY5Y cells. (A) SH‐SY5Y cells were treated with OGD/R for 3 h, and the cells were collected after 24 h of oxygen–glucose reoxygenation. Western blot was used to detect the level of RNF2 and MANF. (B) Quantitation of data as in A (n = 3; ***p < 0.001 vs. Mock; t‐test). (C) Repeat three dishes of cells as in A. (D) Quantitation of data as in C (n = 3; ***p < 0.001 vs. Mock; t‐test). [file CNS-30-e70136-s005.tif]

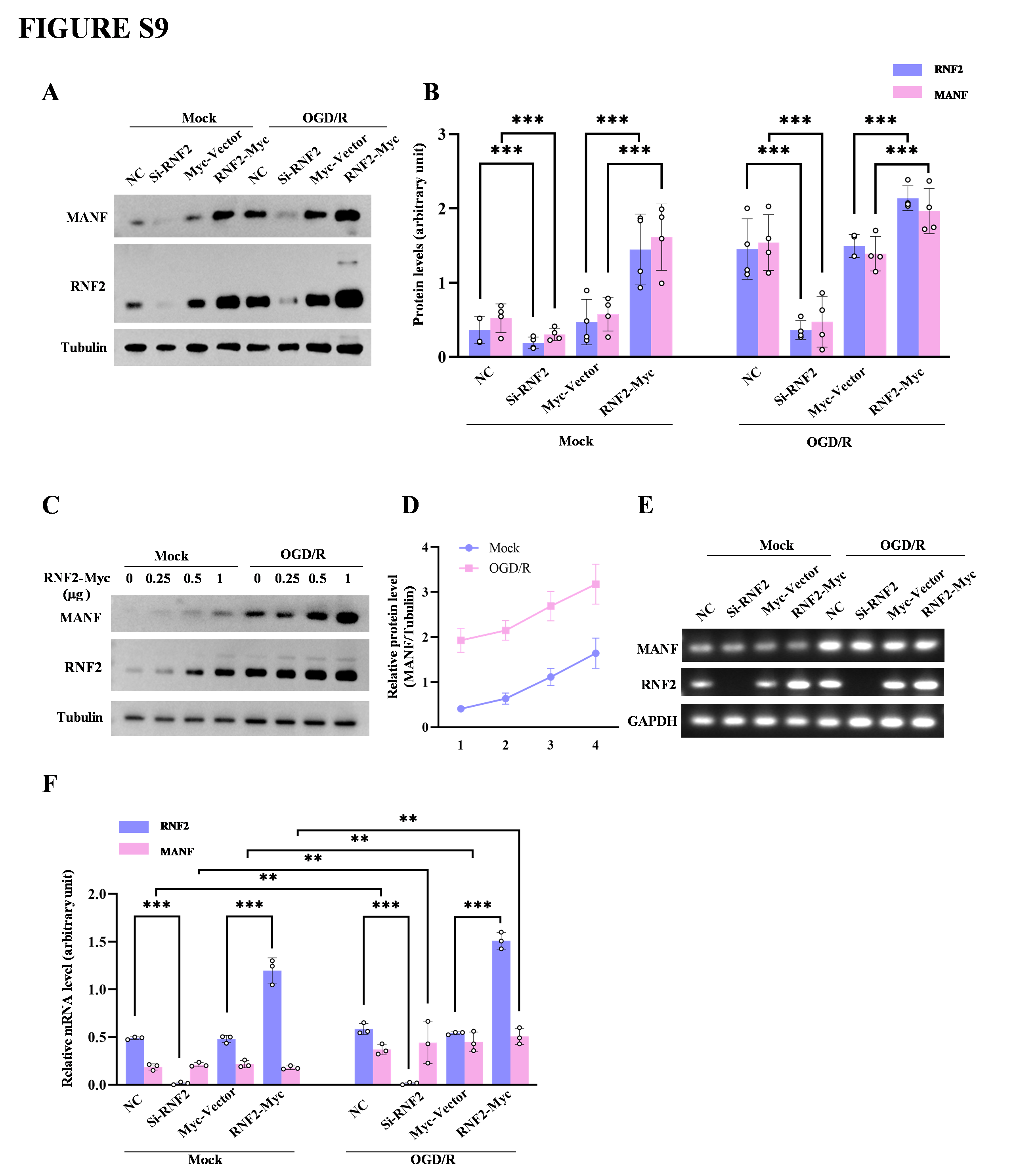

Supplement: Supplementary file 10 — Figure S9. RNF2 regulates the MANF protein level but not the transcription level in SH‐SY5Y cells with OGD/R treatment. SH‐SY5Y cells were transfected with RNF2‐siRNA or RNF2‐Myc and treated with OGD/R at 36 h after transfection. Mock means without OGD/R treatment. The protein levels of RNF2 and MANF were detected with anti‐RNF2 and anti‐MANF antibodies, respectively. Tubulin was used as a loading control. (B) Quantitation of data as in A (n = 4; ***p < 0.001 vs. NC or Myc‐Vector; t‐test). (C) RNF2 upregulates MANF in a dose‐dependent manner. SH‐SY5Y cells were transiently transfected with RNF2‐Myc plasmid (0, 0.25 , 0.5, 1.0 μg). After 36 h of transfection, the cells were treated with OGD/R. The levels of MANF and RNF2 were detected by western blot assay. Tubulin was used as a loading control. (D) Variation curve of the MANF level in C. (E) RNF2 had no effect on the MANF transcription level. SH‐SY5Y cells were transfected with RNF2‐siRNA or RNF2‐Myc for 36 h. The expressions of RNF2 and MANF in SH‐SY5Y cells were detected by using relative quantitative RT‐PCR assay. GAPDH was used as loading control. (F) Quantitation of data as in E (n = 3; **p < 0.01, ***p < 0.001 vs. NC or Myc‐Vector; t‐test). [file CNS-30-e70136-s009.tif]

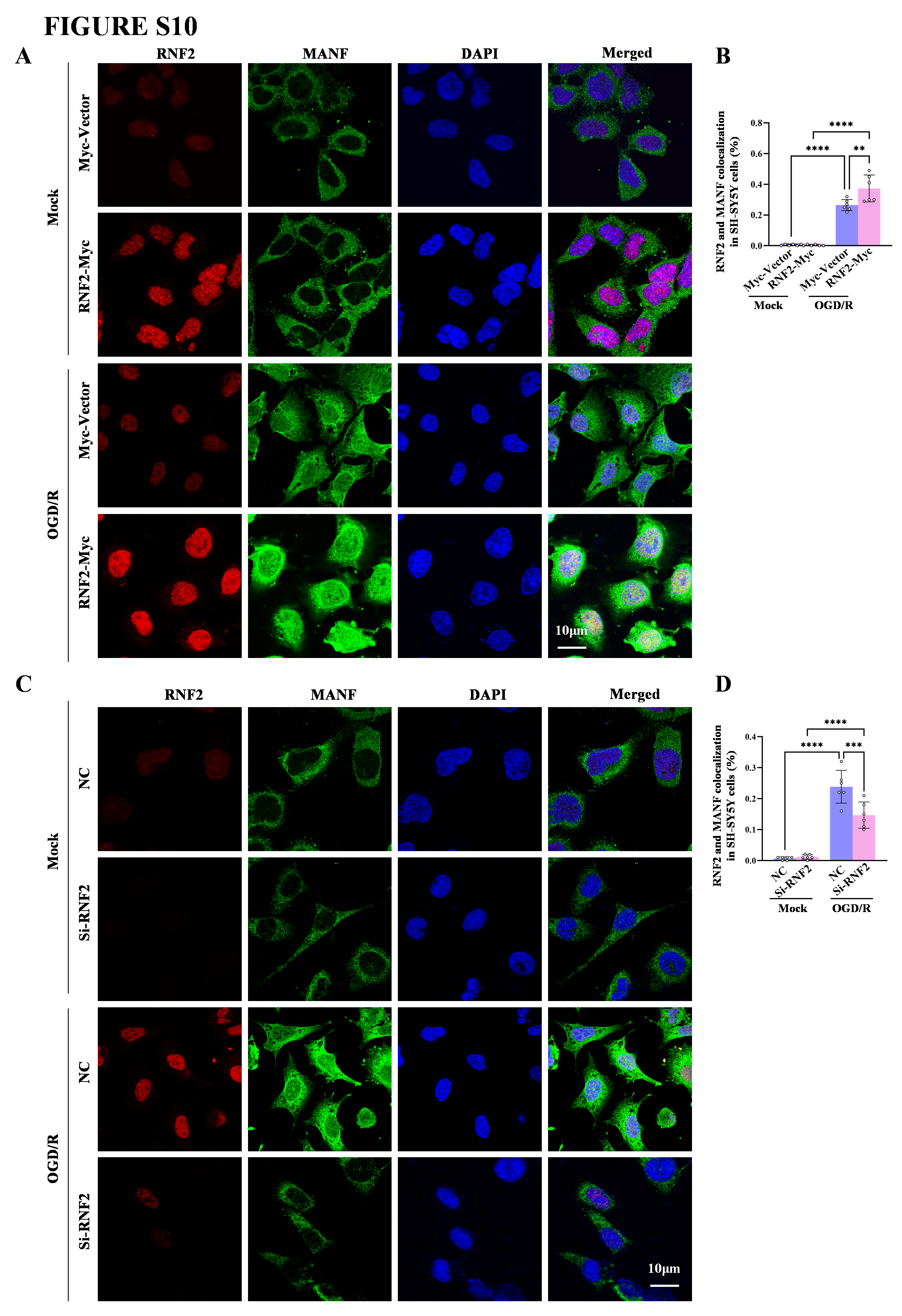

Supplement: Supplementary file 11 — Figure S10. RNF2 interacts with MANF in SH‐SY5Y cells. SH‐SY5Y cells were transfected with RNF2‐Myc or RNF2‐siRNA and treated with OGD/R at 36 h after transfection. (A, C) RNF2 (red) and MANF (green) were detected by immunofluoscent staining in SH‐SY5Y cells. DAPI was used to counterstain the nuclei. The scale bar = 10 μm. (B, D) Quantitation of data as in A and C. Percentage of RNF2 and MANF colocalization cells in A and B. (n = 6; **p < 0.01, ***p < 0.001, ***p < 0.0001 vs. Myc‐Vector or NC; t‐test). [file CNS-30-e70136-s008.tif]

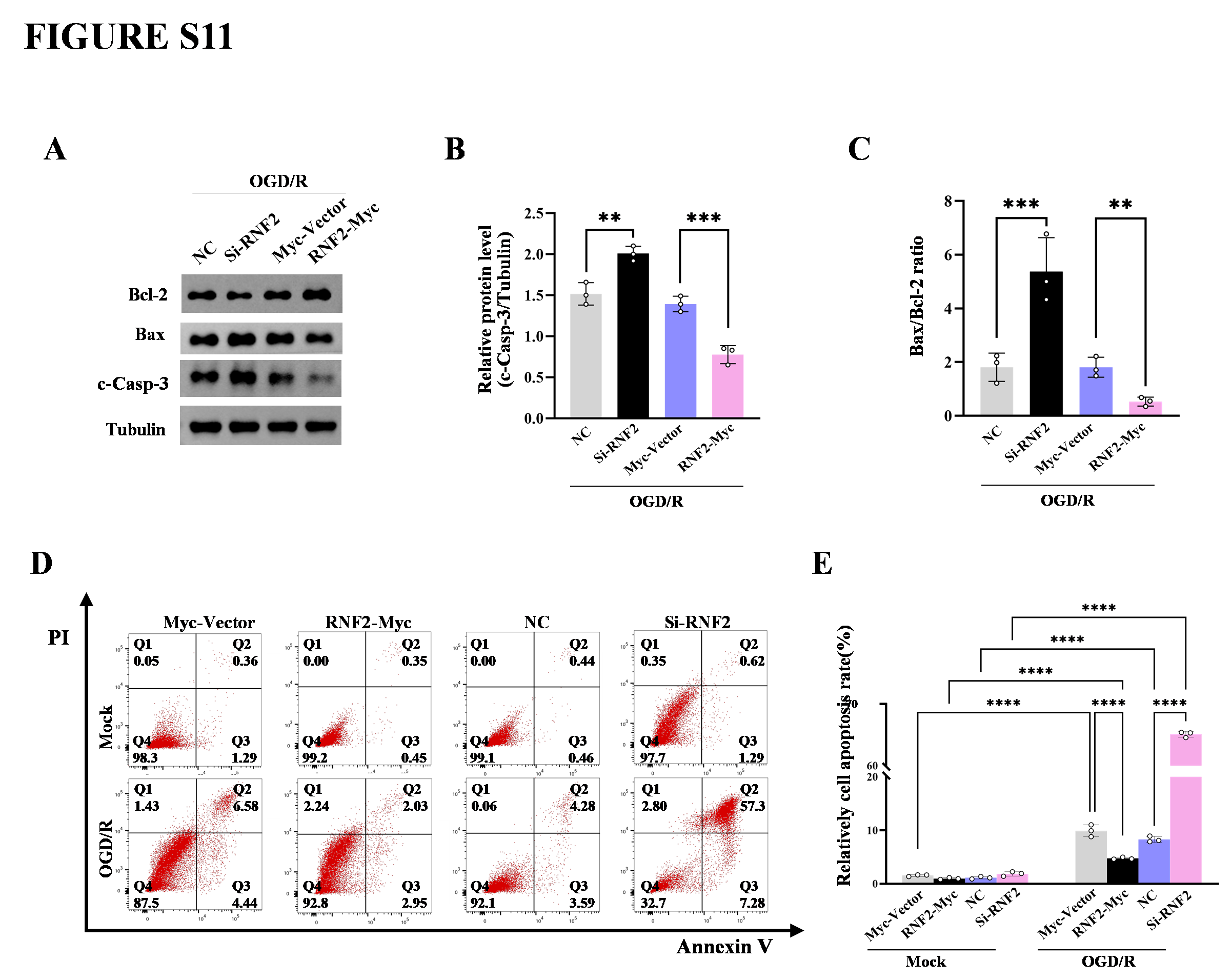

Supplement: Supplementary file 12 — Figure S11. RNF2 inhibits neuronal cells apoptosis dependently on MANF. SH‐SY5Y cells were transfected with RNF2‐Myc or RNF2‐siRNA and treated with OGD/R at 36 h after transfection. (A) The proteins were detected with anti‐bcl‐2, anti‐bax and anti‐cleaved caspases‐3 antibody, respectively. Tubulin was used as a loading control. (B) Quantitation of data as in A (n = 3; **p < 0.01, ***p < 0.001 vs. NC or Myc‐Vector; t‐test). (C) Bax/Bcl‐2 ratio was evaluated as in A (n = 3; **p < 0.01, ***p < 0.001 vs. NC or Myc‐Vector; t‐test). (D) RNF2 overexpression protects against OGD/R‐induced nerve cells apoptosis. SH‐SY5Y cells were transiently transfected with the plasmids and siRNA as indicated. After 36 h of transfection, the cells were treated with OGD/R. (D) Flow cytometry showing apoptotic SH‐SY5Y cells. (E) Quantitation of data as in D (n = 3; ****p < 0.0001 vs. Myc‐Vector or NC; t‐test). [file CNS-30-e70136-s010.tif]

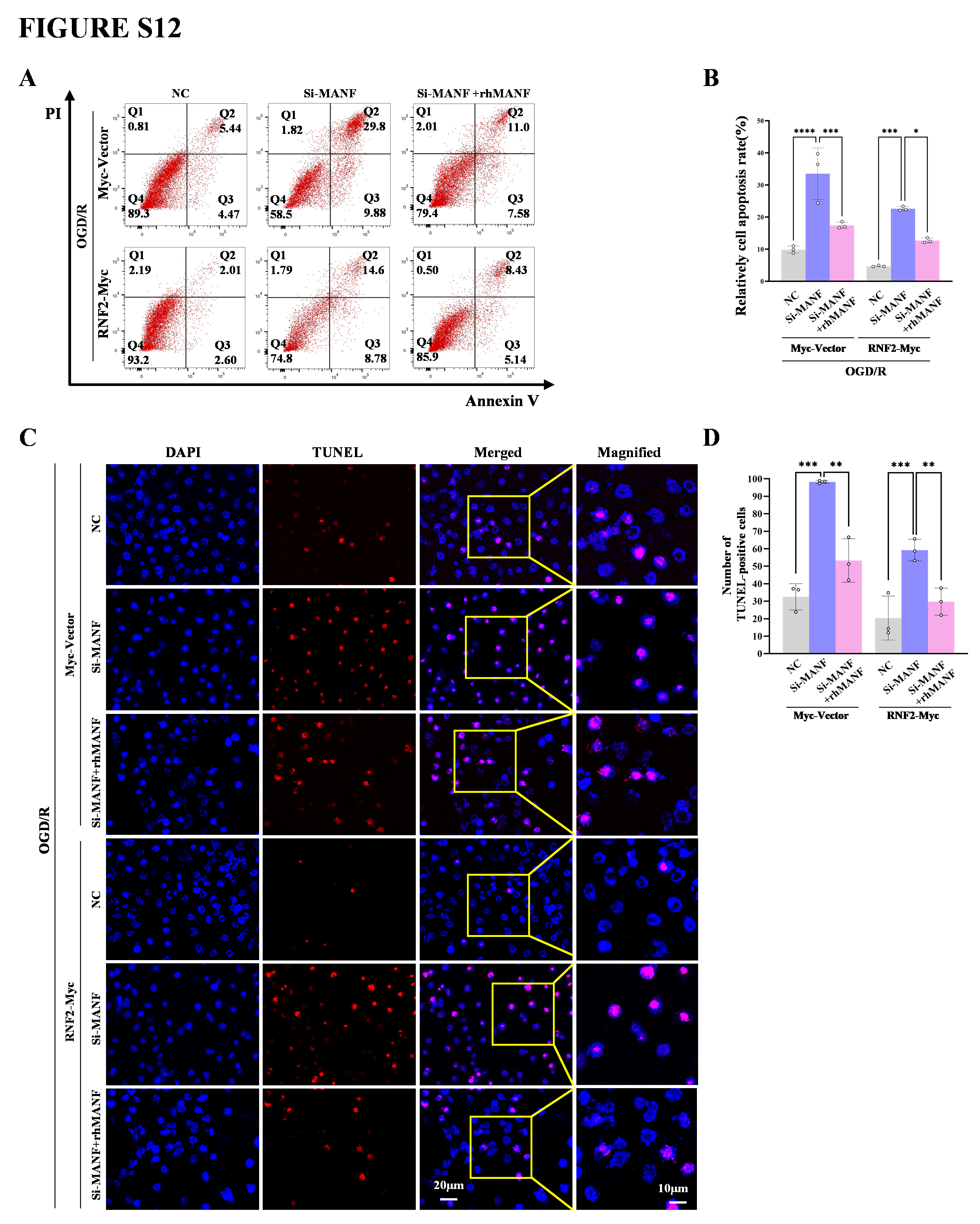

Supplement: Supplementary file 13 — Figure S12. RNF2 inhibits neuronal cells apoptosis dependently on MANF. RNF2‐Myc plasmid was transfected into MANF knockdown SH‐SY5Y cells, or corresponding controls and treated with OGD/R at 36 h posttransfection. SH‐SY5Y cells were treated with rhMANF for 2 h before harvesting. (A) Flow cytometry showing apoptotic SH‐SY5Y cells. (B) Quantitation of data as in A (n = 3; *p < 0.05, ***p < 0.001, ****p < 0.0001; one‐way ANOVA followed by Tukey’s test). (C) Apoptosis as detected by TUNEL assays. Scale bar = 20 μm. Magnified photo scale bar = 10 μm. (D) Quantitation of data as in C (n = 3; **p < 0.01, ***p < 0.001; one‐way ANOVA followed by Tukey’s test). [file CNS-30-e70136-s012.tif]
